# New Natural Oxygenated Sesquiterpenes and Chemical Composition of Leaf Essential Oil from Ivoirian *Isolona dewevrei* (De Wild. & T. Durand) Engl. & Diels

**DOI:** 10.3390/molecules25235613

**Published:** 2020-11-29

**Authors:** Didjour Albert Kambiré, Jean Brice Boti, Thierry Acafou Yapi, Zana Adama Ouattara, Ange Bighelli, Joseph Casanova, Félix Tomi

**Affiliations:** 1 Laboratoire de Constitution et Réaction de la Matière, UFR-SSMT, Université Félix Houphouët-Boigny, Abidjan 01 BP V34, Ivory Coast; dakambire@gmail.com (D.A.K.); jeanbriceboti@hotmail.fr (J.B.B.); acafouth@yahoo.fr (T.A.Y.); 2 Laboratoire de Chimie Bio-Organique et de Substances Naturelles, UFR SFA, Université Nangui Abrogoua, Abidjan 02 BP 801, Ivory Coast; zana1504@yahoo.fr; 3 Laboratoire Sciences Pour l’Environnement, Equipe Chimie et Biomasse, Université de Corse—CNRS, UMR 6134 SPE, Route des Sanguinaires, 20000 Ajaccio, France; bighelli@univ-corse.fr (A.B.); joseph.casanova@wanadoo.fr (J.C.)

**Keywords:** *Isolona dewevrei*, leaf essential oil, (10βH)-1β,8β-oxido-cadin-4-ene, *cis*-germacrene d-8-ol, *trans*-germacrene d-8-ol, cadina-1(10),4-dien-8β-ol

## Abstract

This study aimed to investigate the chemical composition of the leaf essential oil from Ivoirian *Isolona dewevrei*. A combination of chromatographic and spectroscopic techniques (GC(RI), GC-MS and ^13^C-NMR) was used to analyze two oil samples (S1 and S2). Detailed analysis by repetitive column chromatography (CC) of essential oil sample S2 was performed, leading to the isolation of four compounds. Their structures were elucidated by QTOF-MS, 1D and 2D-NMR as (10βH)-1β,8β-oxido-cadin-4-ene (**38**), 4-methylene-(7αH)-germacra-1(10),5-dien-8β-ol (*cis*-germacrene D-8-ol) (**52**), 4-methylene-(7αH)-germacra-1(10),5-dien-8α-ol (*trans*-germacrene D-8-ol) (**53**) and cadina-1(10),4-dien-8β-ol (**56**). Compounds **38**, **52** and **53** are new, whereas NMR data of **56** are reported for the first time. Lastly, 57 constituents accounting for 95.5% (S1) and 97.1% (S2) of the whole compositions were identified. Samples S1 and S2 were dominated by germacrene D (23.6 and 20.5%, respectively), followed by germacrene D-8-one (8.9 and 8.7%), (10βH)-1β,8β-oxido-cadin-4-ene (7.3 and 8.7), 4-methylene-(7αH)-germacra-1(10),5-dien-8β-ol (7.8 and 7.4%) and cadina-1(10),4-dien-8β-ol (7.6 and 7.2%). Leaves from *I. dewevrei* produced sesquiterpene-rich essential oil with an original chemical composition, involving various compounds reported for the first time among the main components. Integrated analysis by GC(RI), GC-MS and ^13^C-NMR appeared fruitful for the knowledge of such a complex essential oil.

## 1. Introduction

*Isolona dewevrei* (De Wild. & T. Durand) Engl. & Diels (synonym: *Monodora dewevrei* De Wild. & T. Durand; genus *Isolona* Engl., Annonaceae) is an evergreen shrub or a tree that can reach 15 m in height. Leaves are narrowly obovate to obovate or elliptic to narrowly elliptic, 10–17 cm long and 4–7 cm wide, with acuminated apex. The inflorescences appear on leafy branches and sometimes on older ones, whereas the fruits are ovoid (6–7 cm long, 4–5 cm in diameter), smooth but very finely ribbed, glabrous, green and become yellow at maturity [1]. The genus *Isolona* consists of 20 species widely distributed in the tropical rain forests of West and Central Africa, and Madagascar. Five species of this genus grow wild in Côte d’Ivoire: *Isolona campanulata*, *I. cooperi*, *I. deightonii*, *I. soubreana* and *I. dewevrei*. *I. cooperi* and *I. campanulata* are used in Ivorian herbal medicine to treat bronchial ailments, skin diseases, hematuria, infertility and to facilitate childbirth [1,2].

Reported studies carried out on solvent extracts of *I. campanulata* and *I. cooperi* have led to the isolation and identification of various alkaloids, sterols and sesquiterpenes [3,4,5]. Concerning the volatile constituents of *Isolona* species, the chemical compositions of essential oils from *I. cooperi* and *I. campanulata* were determined. The main constituents of leaf and stem bark oils from *I. cooperi* were (*Z*)-β-ocimene and γ-terpinene, while the composition of root bark oil was dominated by 5-isopentenylindole and (*E*)-β-caryophyllene [6]. The leaf oil from *I. campanulata* was rich in sesquiterpenes and its composition was dominated either by eudesm-5-en-11-ol or by (*E*)-β-caryophyllene and α-humulene [7]. In previous works, we investigated and reported for the first time the chemical compositions of leaf, root and stem bark essential oils from *I. dewevrei*, dominated by germacrene B/germacrene D and by cyperene, respectively. From the leaf oil, four new compounds were isolated and characterized as 6,12-oxido-germacra-1(10),4,6,11(12)-tetraene, (5αH,10βMe)-6,12-oxido-elema-1,3,6,11(12)-tetraene, germacra-1(10),4,7(11)-trien-6,12-γ-lactone and (1βH,5βH)-6,12-oxido-guaia-6,10(14),11(12)-trien-4α-ol [8]. The structure of germacrene D-8-one, another new natural compound, was also elucidated after isolation from the stem bark essential oil of the plant [9]. 

Continuing the chemical characterization of essential oils of aromatic and medicinal plants from Côte d’Ivoire [10,11,12,13,14], we now report on the chemical composition of the leaf essential oil from *I. dewevrei*, along with isolation and structure elucidation of three new natural sesquiterpenes as well as description of NMR data of a fourth sesquiterpene.

## 2. Results and Discussion

Two leaf essential oil samples (S1 and S2) from *I. dewevrei* growing wild in Côte d’Ivoire were obtained by hydrodistillation of fresh leaves and the yields calculated on a weight basis (*w*/*w*) were 0.105 and 0.121%, respectively. The oil samples were first analyzed by a combination of GC(RI), GC-MS and ^13^C-NMR, following a computerized method developed at the University of Corsica. This method allowed identification of components present at a content as low as 0.4–0.5% and compiled in our laboratory-made ^13^C-NMR spectral data library [15,16]. 

Although various constituents were identified by the mean of the three techniques, several others, some of which were present at appreciable amounts, remained unidentified. Special attention was paid to four of them that belong to the oxygenated sesquiterpene family, according to their apolar and polar retention indices: compounds **38** (retention indices measured on apolar and polar capillary column, respectively (RIa/RIp) = 1534/1853; 7.3 and 8.7%), **52** and **53** (RIa/RIp = 1657/2355; 10.4 and 9.9%) and **56** (RIa/RIp = 1676/2276; 7.6 and 7.2%). Therefore, essential oil sample S2, which had a higher amount (2.9 g) and contained the four unidentified compounds, was subjected to repetitive column chromatography (CC) in order to perform structural elucidation. In parallel, analysis of CC fractions by GC(RI), GC-MS and ^13^C-NMR led to the identification of several minor components. 

### 2.1. Structure Elucidation of Unidentified Compounds

#### 2.1.1. Structure Elucidation of Compound **38**

Compound **38** was obtained with a great degree of purity (GC: 98.7%) in the sub-fraction F4.3.1 (15 mg). The exact mass measured by GC-QTOF-MS was 220.1821 g/mol, corresponding to the empirical formula C_15_H_24_O (calculated mass = 220.1822 g/mol). The ^1^H-NMR, ^13^C-NMR and DEPT spectra were in agreement with the C_15_H_24_O formula, which involved four unsaturation degrees (Table 1) (Supplementary Materials, Figures S1–S8).

^1^H, ^13^C-NMR and DEPT spectra indicated the occurrence of a tri-substituted double bond (C4, 133.78 ppm and C5, 122.89 ppm) and two carbons bearing the oxygen atom (C, 86.26 ppm and CH, 81.09 ppm), belonging to an oxide sub-structure. Taking into account the four unsaturation degrees and this double bond, compound **38** bears a tricyclic structure.

NMR spectra of **38** evidenced an isopropyl group (H11, 1.45 ppm, dsept: 9.3, 6.7 Hz; H12, 0.94 ppm, d: 6.7 Hz) and H13, 0.87 ppm, d: 6.7 Hz), a methyl group (H15, 1.59 ppm, broad s) linked to a sp^2^ quaternary carbon and another one (H14, 1.07 ppm, d: 7.4 Hz) linked to a sp^3^ methine. 

Starting from the methine linked to the oxygen atom (CH, 81.75 ppm, 4.27 ppm, d, 5.2 Hz) the HMBC correlations evidenced the oxa-bicyclo[2.2.1]heptane (oxa norbornane) substructure bearing the isopropyl group on C7 and the methyl group on C10. Correlation plots observed on the COSY spectrum between H7 and H11 on the one hand and between H10 and H14 on the other hand confirmed the position of both substituents on the oxa-norbornane framework. The last four carbons, including two sp^2^ carbons and two sp^3^ carbons, constituted the third cycle, obviously cyclohexenic. HMBC correlation plots allowed the positioning of the cyclohexene sub-structure vs. the oxa-norbornane moiety as well as the position of the methyl group on the double bond. 

Therefore, the molecule under investigation possesses a bicyclo[4.4.0]decane skeleton with an oxide function between C1 and C8, drawing an oxa-norbornane sub-structure that bears a methyl on C10 and an isopropyl group on C7. The second cycle is a cyclohexene with a vinylic methyl on C4. Therefore, this molecule may be considered as a 1,8-oxido-cadin-4-ene. 

Eight stereoisomers may be drawn, four of these display a *cis* stereochemistry of the bicyclo[4.4.0]decane ring junction, the last four display a *trans* stereochemistry of the ring junction. The relative configurations of the ring junction and those of carbons bearing the methyl and isopropyl groups were determined through NOESY correlations. Indeed, the observed correlation between H7 and H14 located the isopropyl group in the exo position vs. the oxa-norbornane sub-structure as well as the methyl 14 in the endo position. In parallel, the NOESY correlation between H6, H11 and H13 corroborated a *cis* junction of the bicyclo[4.4.0]decane framework.

**Figure 1:**
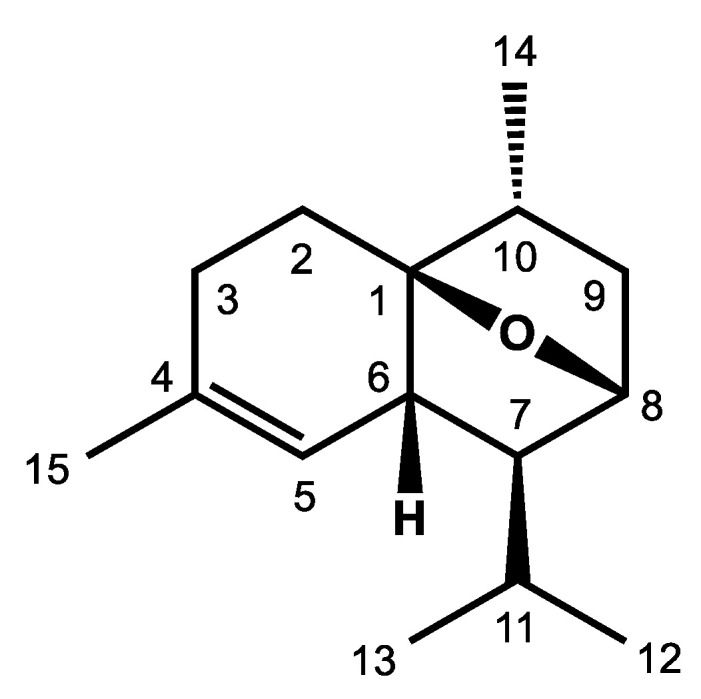
Structure of compound **38**.

The structure of compound **38** was elucidated as (10βH)-1β,8β-oxido-cadin-4-ene, a diastereoisomer of *cis* and *trans*-cadinene ethers, which displays a *trans* junction of the bicyclo[4.4.0]decane skeleton and a different stereochemistry to the isopropyl/methyl groups (Figure 1). 

#### 2.1.2. Structure Elucidation of Compounds **52** and **53**

Sub-fraction F5.3.3 (26 mg) exhibited a single chromatographic peak—(99.4% on GC apolar and polar columns). In contrast, the ^13^C-NMR spectrum of this sub-fraction displayed two series of 15 carbon signals easily distinguishable by their relative intensities (compounds **52** + **53**). The ratio calculated by the mean of protonated carbons’ relative intensities was 7/3 (compounds **52**/**53**). 

The scanning of the chromatographic peak afforded super imposable mass spectra and the exact mass measured was 220.1823 g/mol, corresponding to C_15_H_24_O formula (calculated mass = 220.1822 g/mol). In addition, the two series of ^13^C chemical shifts corresponding to these compounds were very similar. Indeed, each compound displayed six sp^2^ carbon signals, which consisted of two quaternary carbons, three methines and an ethylenic methylene (109.34 and 112.25 ppm, respectively; **52** and **53**). The nine other signals belonged to sp^3^ carbons and each structure of compound was constituted of three methines, of which one carbon linked to an oxygen atom (69.57 and 73.13 ppm, respectively; **52** and **53**), three methylenes and three methyl groups (Table 2) (Supplementary Materials, Figures S9–S17). Therefore, the ^13^C-NMR and DEPT spectra corroborated the C_15_H_24_O formula, which involved four unsaturation degrees. Each compound exhibited six sp^2^ carbons that belonged to three double bonds, which were obviously monocyclic; therefore, they may be considered more precisely as methylene cyclodecadienols and the various observations suggested the presence of two epimers.

Compounds **52** and **53,** which co-eluted on apolar and polar columns, look uneasily separable by chromatographic techniques at our disposal. Therefore, the NMR extraction technique was used on the sub-fraction that contained only the two compounds (99.4%, ratio 7/3) for their structural elucidation [17,18]. This technique consisted of first assigning the ^1^H and ^13^C chemical shifts of each compound, taking into account the relative intensities of their signals and using the HSQC spectrum. Then, the specific correlations of each isomer were plotted on the other 2D-NMR spectra, i.e., COSY, NOESY, HMBC. Lastly, the determination of their respective structure was achieved by using the specific correlations belonging to each compound. 

Concerning the major compound **52**, NMR spectra evidenced an isopropyl group: H11 (1.69 ppm, m), H12 (0.97 ppm, d: 6.7 Hz) and H13 (0.87 ppm, d: 6.7 Hz); as well as a methyl group linked to a sp^2^ quaternary carbon (H14, 1.71 ppm, broad s) and an exocyclic methylene (H15, 4.78 and 4.82, br d: 2.3Hz). Therefore, the molecule contained the cyclodecadiene structure, substituted by a hydroxyl group. In addition, two deshielded methine signals (C8, 69.57 and C7, 57.56 ppm) suggested a first carbon linked to the hydroxyl group and a carbon in α of the previous carbon, probably deshielded by the isopropyl group. This was confirmed by correlations observed on the HMBC spectrum, which evidenced that the isopropyl group was linked to C7. Multiplicity of vinylic proton signals (H5: 5.79 ppm, d, 16.1 Hz; H6: 5.56 ppm, dd, 16.1, 9.8 Hz) demonstrated a CH=CH double bond. According to the correlations observed on the HMBC spectrum, this double bond was located between C7 and the quaternary carbon (C4, 148.76 ppm) of the exocyclic C=CH_2_. This was confirmed by correlation plots observed on the COSY, which also showed two proton groups formed by the sequences H1-H2-H3 and H5-H6-H7-H8-H9. The HMBC correlations of the hydrogens H1, H3 and H9 completed the structure of compound **52** as 4-methylene-germacra-1(10),5-dien-8-ol.

**Figure 2:**
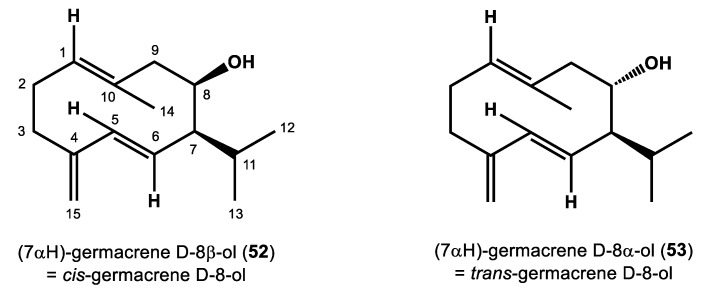
Structure of compounds **52** and **53**.

The (E) stereochemistry of the intracyclic double bonds was evidenced by the value of the coupling constant (16.1 Hz) for C5=C6 and by the occurrence of a correlation plot between H1 and H9 in the NOESY spectrum for C1=C10. The relative stereochemistry of the isopropyl and hydroxyl groups was determined through (i) the values of coupling constants of signals of geminated hydrogens; (ii) NOE spatial correlations observed between various protons. Indeed, H6 appears as a dd (J_H5-H6_ = 16.1 Hz, and J_H6-H7_ = 9.8 Hz). In turn, the signal of H7 is a dt (J_H6-H7_ = 9.8 Hz, J_H7-H11_ = 2.5 Hz and J_H7-H8_ = 2.5 Hz). Assuming that the isopropyl group adopts an equatorial position, H7 is axial and the coupling constant value J_H7-H8_ = 2.5Hz locates H8 in equatorial position. Therefore, H7 and H8 display a *cis* stereochemistry as well as the isopropyl and hydroxyl groups and **52** is *cis*-germacrene D-8-ol. This point is corroborated by the observation in the NOESY spectrum of a correlation plot between H7 and H8, confirming that both protons are in the same side of the molecule. The structure of **52** is elucidated as 4-methylene-(7αH)-germacra-1(10)*E*,5*E*-dien-8β-ol or germacrene-d-8β-ol or *cis*-germacrene-d-8-ol (Figure 2).

Similarly, all correlations observed on HMBC and COSY spectra for the minor component **53** led to the same structure of germacrene D-8-ol. The E stereochemistry of the intracyclic double bonds is evidenced similarly to **52**. Thus, compounds **52** and **53** are epimers. Unfortunately, signals of H7 and H8 appeared as multiplets and therefore they were not useful for stereochemical investigation. Moreover, the NOESY spectrum was not very informative. However, two points may be highlighted; (i) the occurrence of a correlation plot between H8 and H11 (absent in the spectrum of **52**) and (ii) the lack of correlation plot between H7 and H8, which are located in a *trans* antiperiplanar conformation, this plot being observed in the spectrum of **52**. Lastly, considering that the cyclodecadiene moiety adopts a chair-boat-chair conformation, the deshielding (3.5 ppm) of C8 in **53** vs. **52** is in agreement with the axial/equatorial stereochemistry of the hydroxyl group (compared with menthol/neo-menthol, for instance). Compound **53** is named 4-methylene-(7αH)-germacra-1(10),5-dien-8α-ol or (7αH)-germacrene d-8α-ol or *trans*-germacrene d-8-ol.

#### 2.1.3. Structure Elucidation of Compound **56**

Sub-fraction F5.3.1 (19 mg) contained compound **56** (98.3%), with RIs apol/pol = 1676/2276, suggesting an oxygenated sesquiterpene. The electron ionization (EI)-mass spectrum of compound **56** exhibited an *m*/*z* = 220 molecular ion peak (M^•+^) and an M^•+^-18 peak (*m*/*z* = 202), characteristic of a sesquiterpene alcohol. However, no structure proposal emerged from GC-MS analysis with an acceptable fit (commercial MS libraries and home-made MS library). Therefore, structural elucidation was undertaken. 

The measured exact mass was 220.1823 g/mol, corresponding to C_15_H_24_O formula (calculated mass = 220.1822 g/mol). The ^1^H-NMR, ^13^C-NMR and DEPT spectra were in agreement with this formula, which involved four unsaturation degrees. These spectra also confirmed the presence of an alcohol function (C8, 65.54 ppm) (Table 3) (Supplementary Materials, Figures S18–S26). Four sp^2^ carbon signals including three quaternary carbons, involved in two C=C double bonds, were observed. Taking into account the four unsaturation degrees, compound **57** obviously bears a bicyclic structure. The ^1^H-NMR spectrum evidenced an isopropyl group: H11 (1.45 ppm, dsept: 9.3, 6.7 Hz), H12 (0.94 ppm, d: 6.7 Hz) and H13 (0.87 ppm, d: 6.7 Hz); confirmed by the COSY spectrum and two methyl groups linked to sp^2^ quaternary carbons (H14, 1.67 ppm, broad s; H15, 1.69 ppm, broad s). COSY correlations also evidenced two other hydrogen groups formed by the sequences H2-H3 and H5-H6-H7-H8-H9 and they indicated that the isopropyl group was linked to C7.

Correlation plots in the HMBC spectrum allowed the construction of the bicyclic skeleton. For instance, proton H8 geminated to the hydroxyl function correlates with C6, C7, C9 and the ethylenic quaternary carbon C10. Proton H6, located at the ring junction, correlates with C1, C2 and C10 on the one hand and with C4, C5 and C7 on the other hand. Otherwise, protons H14 and H15 correlated with sp^2^ quaternary carbons C10 and C4. Thus, the C14 and C15 methyl groups were linked to C10 and C4, respectively. It is possible to determine the structure of compound **56** as cadina-1(10),4-dien-8-ol.

**Figure 3:**
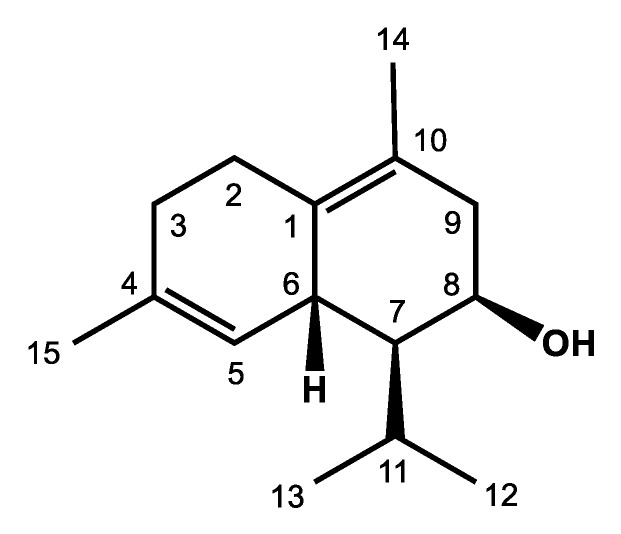
Structure of compound **56**.

The relative stereochemistry of substituents of compound **56** was established through NOESY spatial correlations. Protons H6, H11, H12 and H13 correlated together indicating a *cis* stereochemistry of H6 and the isopropyl group. Similarly, H7 correlated with H8 leading to the *cis* stereochemistry of the isopropyl group and the hydroxyl function. Coupling constants of H6 (11.0 Hz) and H7 (11.0 and 4.3 Hz) are in agreement with NOESY correlations. Therefore, compound **56** is cadina-1(10),4-dien-8β-ol (Figure 3). 

This compound is an epimer of cadina-1(10),4-dien-8α-ol isolated by Weyerstahl et al. [19] from the essential oil of Iranian *Pulicaria gnaphalodes* (Vent.) Boiss. Differences in chemical shifts of both isomers agree with the axial/equatorial stereochemistry of the hydroxyl group, particularly the shielding of C6 due to the γ steric effect of the hydroxyl group. 

However, bibliographic investigations carried out in the literature have indicated that the occurrence of compound **56** has been already mentioned in two studies: 

-In the first one, a compound has been identified in the aerial parts of *Ferula flabelliloba* on the basis of its mass spectrum as being cadina-1(10),4-dien-8β-ol [20]. However, the structure represented by the authors, drawing a *cis* stereochemistry of H6 (hydrogen of the ring junction) and the isopropyl group and a *trans* stereochemistry of the isopropyl and hydroxyl groups, was rather that of the 8α isomer (Figure 3). Moreover, it could be pointed out that the measured retention index (RI) (CP Sil 5 CB) = 1678 [20] fitted with RI (CP Sil 5 CB) = 1680 measured for the 8α isomer [21] and RI (DB1) measured for the 8β isomer (1676; this work). 

-In the second study, cadina-1(10),4-dien-8β-ol was identified by the retention index (RI = 1663, CP Sil 5 CB) and mass spectrum in different organs of *Erigeron annuus* [22]. 

However, to the best of our knowledge, NMR data of cadina-1(10),4-dien-8β-ol were not found in the literature. Therefore, the present study is the first available structural elucidation of that compound.

### 2.2. Chemical Composition of Leaf Essential Oil from I. dewevrei

The chemical composition of two essential oil samples (S1, S2) from wild *I. dewevrei* was determined by a combination of repetitive column chromatography (CC), GC(RI), GC-MS and ^13^C-NMR. In total, fifty-seven components accounting for 95.5 and 97.1% of the composition of the whole oil sample were identified. Compounds **38**, **52** and **53** are reported for the first time, whereas NMR data of **56** are described for the first time. The composition of the two leaf oil samples (S1 and S2) was largely dominated by oxygenated sesquiterpenes (44.1 and 44.9%, respectively) and hydrocarbon sesquiterpenes (41.2 and 40.2%, respectively), the sesquiterpene fraction accounting for 85.3 and 85.1%, respectively (Table 4).

Essential oil samples S1 and S2 displayed close chemical compositions, dominated by germacrene D (23.6 and 20.5%, respectively), followed by germacrene d-8-one (8.9 and 8.7%), (10βH)-1β,8β-oxido-cadin-4-ene (**38**) (7.3 and 8.7%), (7αH)-germacrene d-8β-ol (**52**) (7.8 and 7.4%) and cadina-1(10),4-dien-8β-ol (**56**) (7.6 and 7.2%). Other compounds were also present in both samples at appreciable contents: (*E*)-β-caryophyllene (5.3 and 5.7%), (*E*)-β-ocimene (4.5 and 4.2%) and (*Z*)-β-ocimene (3.4 and 4.5%). 

Investigations carried out on *I. dewevrei* leaf essential oil, using a combination of chromatographic (CC, GC(RI)) and spectroscopic techniques (GC-MS, ^13^C-NMR), led to the identification of fifty-seven constituents accounting for 95.51 and 97.1% of the whole oil samples’ compositions. The two samples were characterized by a similar chemical composition dominated by germacrene D (23.6 and 20.5%, respectively), followed by germacrene d-8-one (8.9 and 8.7%), (10βH)-1β,8β-epoxy-cadina-4-ene (**38**) (7.3 and 8.7%), (7αH)-germacrene d-8β-ol (**52**) (7.8 and 7.4%) and cadina-1(10),4-dien-8β-ol (**56**) (7.6 and 7.2%). Compounds **38**, **52** as well as (7αH)-germacrene D-8α-ol **53** are new natural sesquiterpenes, isolated from sample S2 and fully characterized by QTOF-MS, 1D and 2D-NMR. In addition, cadina-1(10),4-dien-8β-ol (**56**) was also isolated from this oil sample and its NMR data are reported for the first time.

Leaves from *I. dewevrei* produced a sesquiterpene-rich essential oil with an original chemical composition, displaying various compounds that are reported for the first time. The previously reported chemical composition of leaf oil from this species was dominated by germacrene B, (5αH,10βMe)-6,12-oxido-elema-1,3,6,11(12)-tetraene, germacrene D, (*Z*)-β-ocimene, γ-elemene and (*E*)-β-caryophyllene [14]. Thus, qualitative and quantitative differences appeared between the compositions of the previous and the present study. Hence, the chemical variability of the leaf essential oil of *I. dewevrei* should be evaluated by investigating a larger number of oil samples.

## 3. Materials and Methods

### 3.1. Plant Material

The fresh leaves samples (2210 and 2412 g, respectively) were collected on individual *I. dewevrei* trees, which were growing in different ecological conditions, in the Bossématié forest (region of Abengourou, Eastern Côte d’Ivoire, geographical coordinates: 6°26′57.9″ N and 3°28′47.5″ O) in April 2016. Plant material was authenticated by botanists from Centre Suisse de Recherches Scientifiques (CSRS) and Centre National de Floristique (CNF) Abidjan, Côte d’Ivoire. A voucher specimen was deposited at the herbarium of CNF, Abidjan, with the reference LAA 12874.

### 3.2. Essential Oil Isolation and Fractionation

**Scheme 1:**
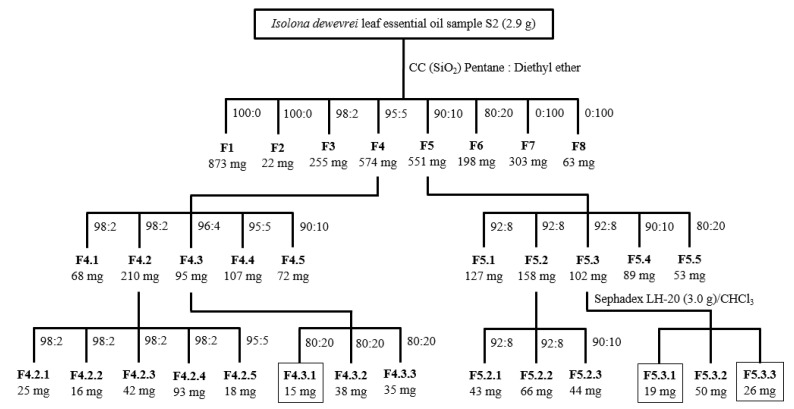
Fractionation process of leaf essential oil sample S2.

The essential oil samples (S1 and S2) were obtained by hydrodistillation of fresh leaves for 3 h using a Clevenger-type apparatus. Yields were calculated from fresh material (*w*/*w*). The oil sample S2 (2.9 g) was repeatedly fractionated by column chromatography (CC) as shown on Scheme 1, using a gradient of solvents, *n*-pentane: diethyl ether of increasing polarity. Silica gel (200–500 μm, 90 g) was used to afford the first eight fractions. Fractions F4 and F5 were again fractionated with silica gel (60–200 μm, 20 g each). Sub-fractions F4.2, F4.3 and F5.2 were then fractionated with silica gel (35–70 μm, 10, 6 and 10 g, respectively). Lastly, sub-fraction F5.3 was submitted to a Sephadex LH-20 column (3.0 g) using chloroform. Compound **38** (98.7%) was the main constituent of sub-fraction F4.3.1. Sub-fraction F5.3.1 contained compound **56** (98.3%) and sub-fraction F5.3.3 contained compounds **52** and **53**, both accounting for 99.4% (ratio 7/3).

### 3.3. Gas Chromatography

Analyses were performed on a Clarus 500 PerkinElmer Chromatograph (PerkinElmer, Courtaboeuf, France), equipped with flame ionization detector (FID) and two fused-silica capillary columns (50 m × 0.22 mm, film thickness 0.25 µm), BP-1 (polydimethylsiloxane) and BP-20 (polyethylene glycol). The oven temperature was programmed from 60 °C to 220 °C at 2 °C/min and then held isothermal at 220 °C for 20 min; injector temperature: 250 °C; detector temperature: 250 °C; carrier gas: helium (0.8 mL/min); split: 1/60; injected volume: 0.5 µL. Retention indices (RI) were determined relative to the retention times of a series of *n*-alkanes (C8–C29) with linear interpolation (« Target Compounds » software from PerkinElmer). The relative response factor (RFF) of each compound was calculated according to the IOFI recommended practice for the use of predicted relative response factors for the rapid quantification of volatile flavoring compounds by GC(FID) [23]. Methyl octanoate was used as an internal reference and the relative proportion of each constituent (expressed in g/100 g) was calculated using the weight of essential oil and reference, peak area and relative response factors (RRF).

### 3.4. Gas Chromatography–Mass Spectrometry in Electron Impact Mode

The essential oil samples and all fractions of chromatography were analyzed with a PerkinElmer TurboMass detector (quadrupole), directly coupled with a PerkinElmer Autosystem XL (PerkinElmer, Courtaboeuf, France), equipped with a Rtx-1 (polydimethylsiloxane) fused-silica capillary column (60 m × 0.22 mm i.d., film thickness 0.25 µm). The oven temperature was programmed from 60 to 230 °C at 2°/min and then held isothermal for 45 min; injector temperature, 250 °C; ion-source temperature, 250 °C; carrier gas, He (1 mL/min); split ratio, 1:80; injection volume, 0.2 µL; ionization energy, 70 eV. The electron ionization (EI) mass spectra were acquired over the mass range 35–350 Da.

### 3.5. Gas Chromatography–High Resolution Mass Spectrometry

High-resolution EI-mass spectra were recorded using an Agilent 7200 GC-QTOF system (Agilent, Santa Clara, CA, USA) equipped with an Agilent J&W, VF-waxMS capillary column (30 m × 0.25 mm; 0.25 μm film thickness). The mass spectrometer was operating at 70 eV with an acquisition rate of 2 GHz over a 35−450 *m*/*z* range, affording a resolution of ∼8000. Injection volume 1 μL; split ratio 1:20; inlet temperature 250 °C, detector temperature 230 °C; column flow (He) 1.2 mL/min; temperature program for oven 60 °C (5 min isotherm) to 240 °C at 5 °C/min, then 10 min isotherm at 240 °C.

### 3.6. Nuclear Magnetic Resonance

All nuclear magnetic resonance (NMR) spectra were recorded on a Bruker AVANCE 400 Fourier transform spectrometer (Bruker, Wissembourg, France) operating at 400.132 MHz for ^1^H and 100.623 MHz for ^13^C, equipped with a 5 mm probe, in CDCl_3_, with all shifts referred to internal TMS. The ^1^H-NMR spectra were recorded with the following parameters: pulse width (PW), 4.3 μs; relaxation delay 1 s and acquisition time 2.6 s for 32 K data table with a spectral width (SW) of 6000 Hz. ^13^C-NMR spectra of the oil samples and fractions of CC were recorded with the following parameters: pulse width = 4 µs (flip angle 45°); acquisition time = 2.7 s for 128 K data table with a spectral width of 25,000 Hz (250 ppm); CPD mode decoupling; digital resolution = 0.183 Hz/pt. Standard pulse sequences from Bruker Topspin^TM^ (Bruker, Wissembourg, France) library were used for two-dimensional spectra. Gradient-enhanced sequences were used for the heteronuclear two-dimensional experiments. Spectra were processed via Mestrelab MestreNOVA software (version 12.0.0-20080).

### 3.7. Identification of Individual Components

Identification of individual components was carried out: (i) by comparison of their GC retention indices (RI) on polar and apolar columns with those of reference compounds [24,25]; (ii) on computer matching against commercial mass spectral libraries [26,27]; (iii) on comparison of the signals in the ^13^C-NMR spectra of the mixtures with those of reference spectra compiled in the laboratory spectral library, with the help of laboratory-made software [15,16]. This method allowed the identification of individual components of the essential oil at content as low as 0.4–0.5%.

### 3.8. Spectral Data

(10βH)-1β,8β-Epoxy-cadina-4-ene (**38**): C_15_H_24_O; ^1^H-NMR (CDCl_3_, 400 MHz) and ^13^C-NMR (CDCl_3_, 100 MHz) data: see Table 1. HREIMS: *m*/*z* 220.1821 (calculated for C_15_H_24_O, 220.1822); EI-MS 70 eV, *m*/*z* (rel. int.): 220(13, M^•+^), 178(15), 177(100), 159(34), 149(43), 135(16), 131(10), 121(19), 119(20), 110(56), 109(11), 107(31), 105(32), 97(67), 95(34), 93(41), 91(41), 81(26), 79(28), 69(78), 67(15), 65(11), 55(46), 53(14), 42(27), 41(58). 

(7αH)-Germacrene d-8β-ol (**52**): C_15_H_24_O; ^1^H-NMR (CDCl_3_, 400 MHz) and ^13^C-NMR (CDCl_3_, 100 MHz) data: see Table 2. HREIMS: *m*/*z* 220.1823 (calculated for C_15_H_24_O, 220.1822); EI-MS 70 eV, *m*/*z* (rel. int.): 220(1, M^•+^), 202(34, M^•+^ − H_2_O), 160(20), 159(100), 146(30), 145(30), 131(50), 121(25), 120(25), 119(50), 117(31), 109(21), 107(45), 105(68), 95(20), 93(65), 92(23), 91(88), 81(53), 80(20), 79(74), 77(47), 69(41), 67(35), 65(20), 55(43), 53(28), 43(61), 41(98).

(7αH)-Germacrene d-α-ol (**53**): C_15_H_24_O; ^1^H-NMR (CDCl_3_, 400 MHz) and ^13^C-NMR (CDCl_3_, 100 MHz) data: see Table 2. HREIMS: *m*/*z* 220.1823 (calculated for C_15_H_24_O, 220.1822); EI-MS 70 eV, *m*/*z* (rel. int.): 220(1, M^•+^), 202(34, M^•+^ − H_2_O), 160(20), 159(100), 146(30), 145(29), 131(48), 121(25), 120(24), 119(49), 117(30), 109(20), 107(44), 105(67), 95(20), 93(63), 92(21), 91(87), 81(51), 80(20), 79(72), 77(47), 69(38), 67(34), 65(19), 55(42), 53(26), 43(60).

Cadina-1(10),4-dien-8β-ol (**56**): C_15_H_24_O; ^1^H-NMR (CDCl_3_, 400 MHz) and ^13^C-NMR (CDCl_3_, 100 MHz) data: see Table 3. HREIMS: *m*/*z* 220.1823 (calculated for C_15_H_24_O, 220.1822); EI-MS 70 eV, *m*/*z* (rel. int.): 220(1, M^•+^), 202(12, M^•+^ − H_2_O), 187(28), 174(7), 160(13), 159(100), 146(6), 145(11), 144(7), 134(10), 131(15), 129(6), 121(6), 119(21), 117(8), 115(6), 107(6), 105(19), 93(9), 91(19), 79(8), 77(10), 55(7), 43(9), 41(15).

## Figures and Tables

**Table 1:** NMR data of compound **38**.

Compound 38							
C	δ ^13^C (ppm)	DEPT	δ ^1^H (ppm)	Multiplicity (J (Hz))	COSY ^1^H–^1^H	HMBC H → C	NOESY ^1^H–^1^H
1	86.63	C	-	-	-	-	-
2	30.71	CH_2_	a 2.15	m	2b, 3a, 3b	1, 3, 4, 6, 10	2b, 3a
			b 2.29	m	2a, 3a, 3b	1, 3, 4, 6, 10	2a, 3b
3	30.13	CH_2_	a 2.00	m	2a, 2b, 3b	1, 2, 4, 5, 15	2a, 3b
			b 2.17	m	2b, 3a, 3b	1, 2, 4, 5, 15	2b, 3a, 15
4	133.78	C	-	-	-	-	-
5	122.89	CH	5.57	quint (1.5)	6	1, 4, 6, 7, 15	6, 13, 15
6	51.98	CH	2.25	m	5, 7	1, 2, 4, 5, 7, 10	5, 11, 13
7	54.01	CH	1.16	t (9.3)	6, 8, 11	5, 6, 8, 11, 12, 13	2b, 8, 9a, 14
8	81.75	CH	4.27	d (5.2)	7, 9a, 9b	6, 7, 9, 10, 11	7, 9a, 14
9	43.42	CH_2_	a 1.02	dd (11.0, 3.8)	8, 9b, 10	7, 8, 10, 14	9b, 8, 7, 14
			b 2.21	dd (11.0, 5.2)	8, 9a, 10	1, 7, 8, 10, 14	9a, 10
10	41.18	CH	2.02	m	14, 9a, 9b	1, 2, 6, 8, 9, 14	9b, 14
11	33.27	CH	1.45	dsept (9.3, 6.7)	7, 12, 13	6, 7, 8, 12, 13	6, 12, 13
12	21.81	CH_3_	0.94	d (6.7)	11	7, 11, 13	11, 13
13	19.82	CH_3_	0.87	d (6.7)	11	7, 11, 12	5, 6, 11, 12
14	19.71	CH_3_	1.07	d (7.4)	10	1, 9, 10	7, 8, 9a, 10
15	22.65	CH_3_	1.59	br s	-	3, 4, 5	3b, 5

**Table 2:** NMR data of compounds **52** and **53**.

Compound 52								Compound 53			
C	δ ^13^C (ppm)	DEPT	δ ^1^H (ppm)	Multiplicity (J (Hz))	COSY ^1^H–^1^H	HMBC H → C	NOESY ^1^H–^1^H	δ ^13^C (ppm)	δ ^1^H (ppm)	Multiplicity (J (Hz))	NOESY ^1^H–^1^H
1	132.27	CH	5.14	br dd (10.7, 4.8)	2a, 2b	2, 9, 10, 14	2b, 6, 9b	130.27	5.63	br dd (10.6, 5.0)	2b, 6, 9b
2	29.36	CH_2_	a 1.99	m	1, 2b, 3a, 3b	1, 3, 4, 5, 10, 15	3b, 14	30.58	a 2.16	m	3b, 14
			b 2.46	m	1, 2a, 3a, 3b	1, 3, 4, 5, 10, 15	1, 3a, 15b		b 2.19	m	1, 3a, 15b
3	34.61	CH_2_	a 2.13	m	1, 2a, 2b	1, 2, 4, 5, 15	2b, 6, 15b	32.26	a 2.22	m	2b, 6, 15b
			b 2.48	m	1, 2a, 2b	1, 2, 4, 5, 15	2a, 5, 8, 14		b 2.46	m	2a, 5, 14
4	148.76	C	-	-	-	-	-	148.79	-	-	-
5	137.36	CH	5.79	d (16.1)	6	3, 4, 6, 7, 15	3b, 7, 8, 14	127.64	6.06	d (16.3)	3b, 7, 14
6	130.18	CH	5.56	dd (16.1, 9.8)	5, 7	4, 5, 7, 8, 11	1, 2b, 9b, 11, 13	134.81	5.93	dd (16.3, 9.8)	1, 2b, 9b, 11, 13
7	57.56	CH	1.93	dt (9.8, 2.5)	6, 8, 11	5, 6, 8,11, 12, 13	5, **8**, 12, 13, 14	53.96	2.12	m	5, 12, 13, 14
8	69.57	CH	4.12	m	7, 9a, 9b	6, 7, 9, 10, 11	3b, 5, **7**, 14	73.13	4.09	m	9a, 13
9	47.28	CH_2_	a 2.39	dd (14.0, 2.3)	8	1, 7, 8, 10, 14	9b, 14	45.68	a 2.02	dd (14.2, 4.0)	8, 9b, 14
			b 2.56	dd (14.0, 5.3)	8	1, 7, 8, 10, 14	1, 6, 9a, 11		b 2.70	dd (14.2, 6.8)	1, 6, 9a, 11
10	132.55	C	-	-	-	-	-	134.16	-	-	-
11	28.47	CH	1.69	m	7, 12, 13	6, 7, 8, 12, 13	6, 9b, 12, 13	27.44	1.97	m	6, 9b, 12, 13
12	20.52	CH_3_	0.97	d (6.7)	11	7, 11, 13	7, 11, 13	20.64	0.99	d (6.8)	7, 11, 13
13	21.58	CH_3_	0.87	d (6.7)	11	7, 11, 12	6, 7, 11, 12	21.86	0.94	d (6.8)	6, 8, 11, 12
14	19.34	CH_3_	1.71	br s	-	1, 2, 8, 9, 10	2a, 3b, 5, 7, 8, 9a	19.53	1.44	br s	2a, 3b, 5, 7, 9a
15	109.34	CH_2_	a 4.78	br d (2.3)	15b	2, 3, 4, 5	3b, 5, 15b	112.25	4.71	br d (2.2)	3b, 5, 15b
			b 4.82	br d (2.3)	15a	2, 3, 4, 5	2b, 3a, 15a		4.88	br d (2.2)	2b, 3a, 15a

**Table 3:** NMR data of compound **56**.

C	δ ^13^C (ppm)	DEPT	δ ^1^H (ppm)	Multiplicity (J (Hz))	COSY ^1^H–^1^H	HMBC H → C	NOESY ^1^H–^1^H ^a^
1	130.31	C	-	-	-	-	-
2	26.70	CH_2_	a 1.99	m	2b, 3	1, 3, 4, 6, 10	2b, 3
			b 2.74	ddd (12.2, 3.6, 3.1)	2a, 3	1, 3, 4, 6, 10	2a, 3, 14
3	32.05	CH_2_	a 2.04	m	2a, 2b	1, 2, 4, 5, 15	2a, 2b, 15
4	134.83	C	-	-	-	-	-
5	123.99	CH	5.45	m (1.5)	6	1, 4, 6, 7, 15	6, 15
6	34.93	CH	2.86	br d (11.0)	5, 7	1, 2, 4, 5, 7, 10	5, 9b, 11, 13
7	48.03	CH	1.15	br dd (11.0, 4.3)	6, 8, 11	5, 6, 8, 11, 12, 13	8, 9a, 14
8	65.54	CH	4.17	m	7, 9a, 9b	6, 7, 9, 10, 11	7, 9a, 14
9	41.81	CH_2_	a 2.04	m	8, 9b	1, 7, 8, 10, 14	9b, 8, 7, 14
			b 2.30	dd (17.3, 4.1)	8, 9a	1, 7, 8, 10, 14	9a, 6, 11, 12
10	119.79	C	-	-	-	-	-
11	27.14	CH	2.10	dsept (7.0, 4.1)	7, 12, 13	6, 7, 8, 12, 13	6, 12, 13
12	18.50	CH_3_	1.04	d (7.0)	11	7, 11, 13	9b, 11, 13
13	21.79	CH_3_	1.05	d (7.0)	11	7, 11, 12	6, 11, 12
14	18.74	CH_3_	1.67	br s	-	1, 9, 10	2b, 8, 7
15	23.62	CH_3_	1.69	br s	-	3, 4, 5	3, 5

^a^ Most relevant NOE correlations.

**Table 4:** Chemical composition of leaf essential oil from *Isolona dewevrei*.

N°	Compounds	RIa	RIp	RFF	S1 (%)	S2 (%)	Identification
1	α-Thujene	923	1016	0.765	tr	0.1	RI, MS
2	α-Pinene	931	1013	0.765	0.1	0.1	RI, MS
3	Sabinene	965	1120	0.765	0.1	0.4	RI, MS, ^13^C-NMR
4	β-Pinene	970	1109	0.765	0.1	0.2	RI, MS, *^13^C-NMR*
5	Myrcene	981	1158	0.765	0.3	0.3	RI, MS, *^13^C-NMR*
6	α-Terpinene	1009	1178	0.765	0.1	0.1	RI, MS
7	*p*-Cymene	1012	1268	0.698	tr	0.1	RI, MS, ^13^C-NMR
β	Limonene	1021	1199	0.765	1.1	1.1	RI, MS, ^13^C-NMR
9	(*Z*)-β-Ocimene	1025	1230	0.765	3.4	4.5	RI, MS, ^13^C-NMR
10	(*E*)-β-Ocimene	1036	1247	0.765	4.5	4.2	RI, MS, ^13^C-NMR
11	γ-Terpinene	1048	1242	0.765	0.2	0.2	RI, MS, *^13^C-NMR*
12	Linalool	1083	1543	0.869	tr	0.1	RI, MS
13	*allo*-Ocimene	1117	1370	0.765	0.1	0.2	RI, MS, *^13^C-NMR*
14	Terpinen-4-ol	1161	1597	0.869	-	0.1	RI, MS
15	Geraniol	1233	1843	0.869	0.1	0.2	RI, MS, *^13^C-NMR*
16	Geranial	1244	1740	0.887	0.1	0.1	RI, MS, ^13^C-NMR
17	δ-Elemene	1334	1464	0.751	tr	0.5	RI, MS, ^13^C-NMR
18	α-Cubebene	1347	1452	0.751	0.1	0.1	RI, MS
19	α-Copaene	1374	1485	0.751	0.9	0.7	RI, MS, ^13^C-NMR
20	β-Elemene	1385	1583	0.751	1.6	1.7	RI, MS, ^13^C-NMR
21	(*E*)-β-Caryophyllene *	1416	1589	0.751	5.3	5.7	RI, MS, ^13^C-NMR
22	α-Santalene *	1416	1565	0.751	0.1	0.3	RI, MS, *^13^C-NMR*
23	γ-Elemene #	1426	1630	0.751	tr	0.5	RI, MS, ^13^C-NMR
24	(*E*)-β-Farnesene	1446	1660	0.751	0.1	0.1	RI, MS
25	α-Humulene	1448	1662	0.751	1.7	1.3	RI, MS, ^13^C-NMR
26	α-Curcumene	1469	1766	0.707	tr	tr	RI, MS, ^13^C-NMR
27	γ-Muurolene	1471	1683	0.751	0.3	0.3	RI, MS, *^13^C-NMR*
28	Germacrene D	1474	1700	0.751	23.6	20.5	RI, MS, ^13^C-NMR
29	*trans*-β-Bergamotene	1478	1676	0.751	tr	0.2	RI, MS, *^13^C-NMR*
30	β-Selinene	1484	1710	0.751	0.1	tr	RI, MS
31	Bicyclogermacrene	1489	1721	0.751	1.8	1.6	RI, MS, ^13^C-NMR
32	α-Selinene	1493	1717	0.751	0.2	0.2	RI, MS, ^13^C-NMR
33	β-Bisabolene	1500	1719	0.751	0.2	0.2	RI, MS, ^13^C-NMR
34	δ-Cadinene	1512	1753	0.751	2.5	2.4	RI, MS, ^13^C-NMR
35	*cis*-Lanceol	1517	2087	0.819	0.9	0.7	RI, MS, ^13^C-NMR
36	(*Z*)-γ-Bisabolene	1521	1721	0.751	1.4	1.5	RI, MS, ^13^C-NMR
37	*trans*-Sesquisabinene hydrate	1530	1984	0.819	tr	0.1	RI, MS
38	(10βH)-1β,8β-Oxido-cadin-4-ene	1534	1853	0.830	7.3	8.7	QTOF-MS, 1D, 2D-NMR
39	β-Elemol	1536	2077	0.819	tr	0.2	RI, MS, *^13^C-NMR*
40	(*E*)-Nerolidol	1547	2034	0.819	0.5	1.1	RI, MS, ^13^C-NMR
41	Germacrene B #	1549	1818	0.751	1.3	2.4	RI, MS, ^13^C-NMR
42	*cis*-Sesquisabinene hydrate	1562	2079	0.819	0.3	0.2	RI, MS, ^13^C-NMR
43	Caryophyllene oxide	1567	1973	0.830	0.1	0.2	RI, MS, ^13^C-NMR
44	Germacrene D-8-one	1584	2066	0.841	8.9	8.7	RI, MS, ^13^C-NMR
45	Humulene oxide II	1597	2042	0.830	0.4	0.2	RI, MS, ^13^C-NMR
46	Alismol	1609	2245	0.830	0.1	0.3	RI, MS, *^13^C-NMR*
47	γ-Eudesmol	1620	2172	0.819	1.2	1.2	RI, MS, ^13^C-NMR
48	δ-Cadinol	1626	2174	0.819	0.1	0.2	RI, MS, *^13^C-NMR*
49	Muurola-4,10(14)-dien-8β-ol	1629	2186	0.830	3.2	2.9	RI, MS, ^13^C-NMR
50	α-Cadinol	1634	2231	0.819	0.6	0.6	RI, MS, ^13^C-NMR
51	β-Bisabolol	1653	2144	0.819	0.2	0.2	RI, MS, *^13^C-NMR*
52	(7αH)-Germacrene D-8β-ol *	1657	2355	0.819	7.8	7.4	QTOF-MS, 1D, 2D-NMR
53	(7αH)-Germacrene D-αβ-ol *	1657	2355	0.819	2.6	2.5	QTOF-MS, 1D, 2D-NMR
54	α-Bisabolol	1664	2208	0.819	1.4	1.5	RI, MS, ^13^C-NMR
55	*epi*-α-Bisabolol	1667	2214	0.819	0.1	tr	RI, MS, ^13^C-NMR
56	Cadina-1(10),4-dien-8β-ol	1676	2276	0.819	7.6	7.2	QTOF-MS, 1D, 2D-NMR
57	Cadina-4,10(14)-dien-8β-ol	1678	2280	0.830	0.8	0.8	RI, MS, ^13^C-NMR
	Hydrocarbon monoterpenes				10.0	11.5	
	Oxygenated monoterpenes				0.2	0.5	
	Hydrocarbon sesquiterpenes				41.2	40.2	
	Oxygenated sesquiterpenes				44.1	44.9	
	Total				95.5	97.1	

Order of elution and percentages are given on an apolar column (BP-1), except components with an asterisk (*), where percentages are taken on a polar column (BP-20). (#) Thermolabile compound, percentage evaluated by a combination of GC-FID and ^13^C-NMR data [7]. RIa, RIp: retention indices measured on apolar and polar capillary column, respectively. RRF: relative response factors calculated using methyl octanoate as internal standard. The relative proportions of constituent are expressed in g/100 g. tr: traces level (<0.05%). *^13^C-NMR*: compounds identified by NMR in the essential oil samples and obvious in at least one fraction of chromatography; *^13^C-NMR* (*italic*): compounds identified by NMR in fractions of chromatography.

## Supplementary Materials

The following are available online, Figures S1–S24: 1D, 2D-NMR and EI-mass spectra of (10βH)-1β,8β-oxido-cadin-4-ene (**38**), (7αH)-Germacrene D-8β-ol (**52**), (7αH)-Germacrene D-8α-ol (**53**) and Cadina-1(10),4-dien-8β-ol (**56**).
