# Supplementary material for: New Natural Oxygenated Sesquiterpenes and Chemical Composition of Leaf Essential Oil from Ivoirian Isolona dewevrei (De Wild. & T. Durand) Engl. & Diels
Source: Molecules. 2020 Nov 29;25(23):5613. doi: 10.3390/molecules25235613 (PMC7731318; doi:10.3390/molecules25235613)

Supplementary Materials:

New Natural Oxygenated Sesquiterpenes and Chemical Composition of Leaf Essential Oil from Ivoirian Isolona dewevrei (De Wild. & T. Durand) Engl. & Diels.

Didjour Albert Kambiré [1], Jean Brice Boti [1], Thierry Acafou Yapi [1], Zana Adama Ouattara [2], Ange Bighelli [3], Joseph Casanova [3] and Félix Tomi [3*]

[1] Laboratoire de Constitution et Réaction de la Matière, UFR-SSMT, Université Félix Houphouët-Boigny, BP V34 Abidjan, Côte d'Ivoire; dakambire@gmail.com (D.A.K.); acafouth@yahoo.fr (T.A.Y.)

[2] Laboratoire de Chimie Bio-Organique et de Substances Naturelles, UFR SFA, Université Nangui Abrogoua, 02 BP 801 Abidjan 02, Côte d'Ivoire; zana1504@yahoo.fr (Z.A.O.)

[3] Université de Corse – CNRS, UMR 6134 SPE, Laboratoire Sciences pour l'Environnement, Equipe Chimie et Biomasse, Route des Sanguinaires, 20000 Ajaccio, France; bighelli@univ-corse.fr (A.B.); felix.tomi@univ-corse.fr (F.T.); joseph.casanova@wanadoo.fr (J.C.)

* Correspondence: tomi_f@univ-corse.fr

Received: date; Accepted: date; Published: date

Abstract: This study aimed to investigate the chemical composition of the leaf essential oil from Ivoirian *Isolona dewevrei*. Combination of chromatographic and spectroscopic techniques [GC(RI), GC-MS and ^{13}C-NMR] was used to analyze two oil samples (S1 and S2). Detailed analysis by repetitive column chromatography (CC) of essential oil sample S2 was performed, leading to the isolation of four compounds. Their structures were elucidated by QTOF-MS, 1D and 2D-NMR as (10βH)-1β,8β-oxido-cadin-4-ene (**38**), 4-methylene-(7αH)-germacra-1(10),5-dien-8β-ol (*cis*-germacrene D-8-ol) (**52**), 4-methylene-(7αH)-germacra-1(10),5-dien-8α-ol (*trans*-germacrene D-8-ol) (**53**) and cadina-1(10),4-dien-8β-ol (**56**). Compounds **38**, **52** and **53** are new, whereas NMR data of **56** are reported for the first time. Lastly, 57 constituents accounting for 95.5% (S1) and 97.1%(S2) of the whole compositions were identified. Samples S1 and S2 were dominated by germacrene D (23.6 and 20.5%, respectively), followed by germacrene D-8-one (8.9 and 8.7%), (10βH)-1β,8β-oxido-cadin-4-ene (7.3 and 8.7), 4-methylene-(7αH)-germacra-1(10),5-dien-8β-ol (7.8 and 7.4%) and cadina-1(10),4-dien-8β-ol (7.6 and 7.2%). Leaves from *I. dewevrei* produced sesquiterpene-rich essential oil with original chemical composition, involving various compounds reported for the first time among the main components. Integrated analysis by GC(RI), GC-MS and ^{13}C-NMR appeared fruitful for the knowledge of such complex essential oil.

Keywords: *Isolona dewevrei*; leaf essential oil; (10βH)-1β,8β-oxido-cadin-4-ene; *cis*-germacrene D-8-ol; *trans*-germacrene D-8-ol; cadina-1(10),4-dien-8β-ol.

.

Figure S1: ^{1}H NMR spectrum of **38** in $CDCl_3$ (400 MHz).

Figure S2: ^{13}C NMR spectrum of **38** in $CDCl_3$ (100 MHz).

Figure S3: DEPT 135 NMR spectrum of **38** in $CDCl_3$ (100 MHz).

Figure S4: DEPT 90 NMR spectrum of **38** in $CDCl_3$ (100 MHz).

Figure S5: HSQC spectrum of **38** in $CDCl_3$.

Figure S6: COSY spectrum of **38** in $CDCl_3$.

Figure S7: HMBC spectrum of **38** in $CDCl_3$.

Figure S8: NOESY spectrum of **38** in $CDCl_3$.

Figure S9: ^{1}H NMR spectrum of **52** and **53** in $CDCl_3$ (400 MHz).

Figure S10: ^{13}C NMR spectrum of **52** and **53** in $CDCl_3$ (100 MHz).

Figure S11: DEPT 135 NMR spectrum of **52** and **53** in $CDCl_3$ (100 MHz).

Figure S12: DEPT 90 NMR spectrum of **52** and **53** in $CDCl_3$ (100 MHz).

Figure S13: HSQC spectrum of **52** and **53** in $CDCl_3$.

Figure S14: COSY spectrum of **52** and **53** in $CDCl_3$.

Figure S15: HMBC spectrum of **52** and **53** in $CDCl_3$.

Figure S16: NOESY spectrum of **52** and **53** in $CDCl_3$.

Figure S17: EI-MS spectra of **52** and **53**.

Figure S18: ^{1}H NMR spectrum of **56** in $CDCl_3$ (400 MHz).

Figure S19: ^{13}C NMR spectrum of **56** in $CDCl_3$ (100 MHz).

Figure S20: DEPT 135 NMR spectrum of **56** in $CDCl_3$ (100 MHz).

Figure S21: DEPT 90 NMR spectrum of **56** in $CDCl_3$ (100 MHz).

Figure S22: HSQC spectrum of **56** in $CDCl_3$.

Figure S23: COSY spectrum of **56** in $CDCl_3$.

Figure S24: HMBC spectrum of **56** in $CDCl_3$.

Figure S25: NOESY spectrum of **56** in $CDCl_3$.

Figure S26: EI-MS spectrum of **56**.

Figure S1: ^{1}H NMR spectrum of **38** in $CDCl_3$ (400 MHz).

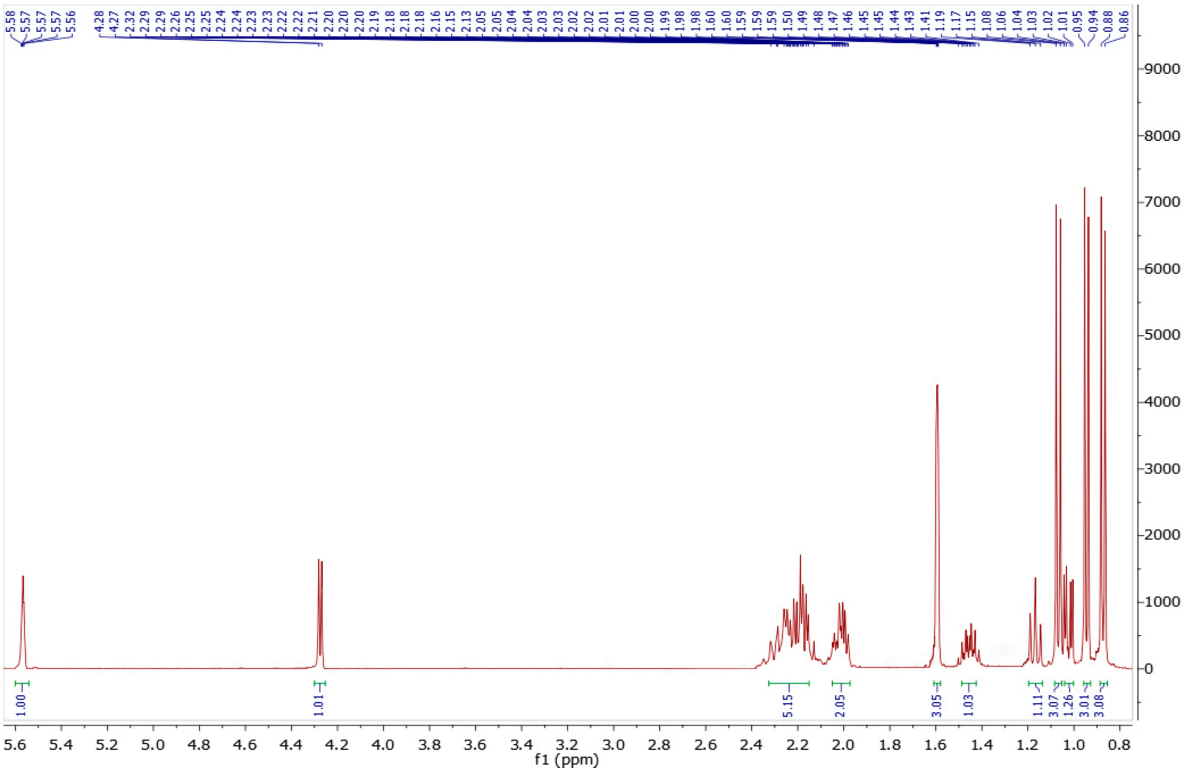

Figure S2: ^{13}C NMR spectrum of **38** in $CDCl_3$ (100 MHz).

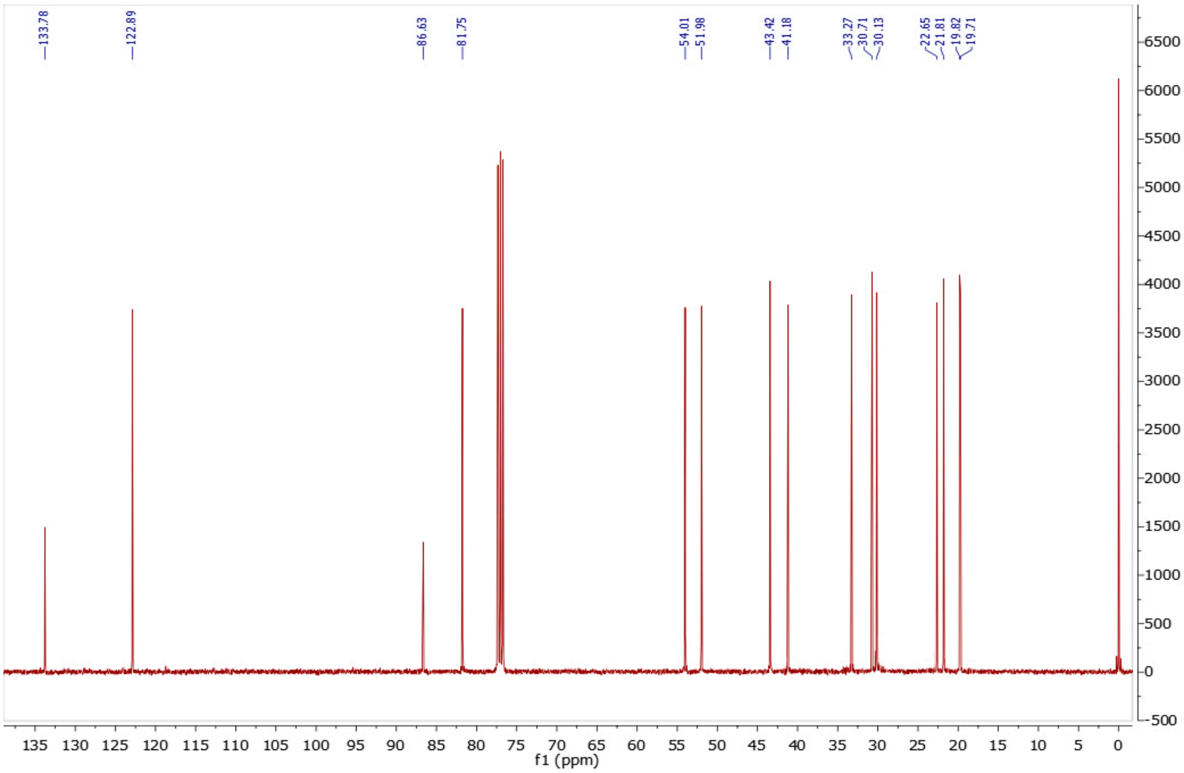

Figure S3: DEPT 135 NMR spectrum of **38** in $CDCl_3$ (100 MHz).

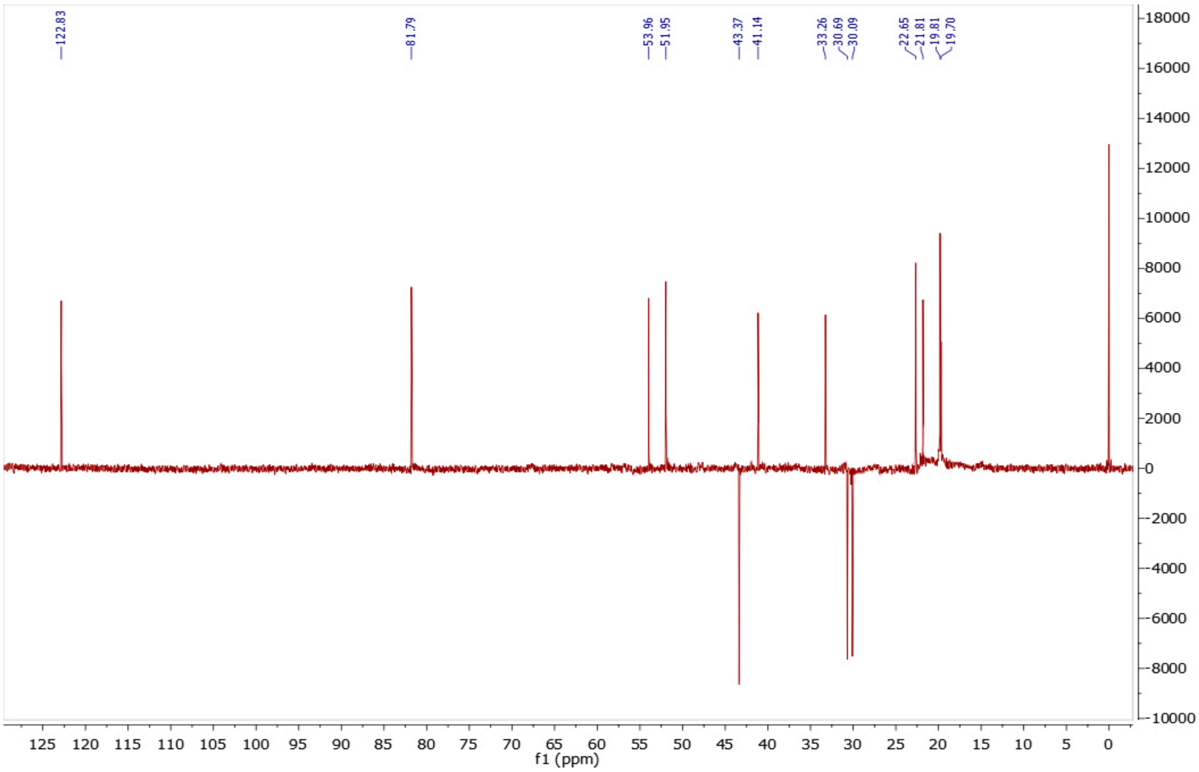

Figure S4: DEPT 90 NMR spectrum of **38** in $CDCl_3$ (100 MHz).

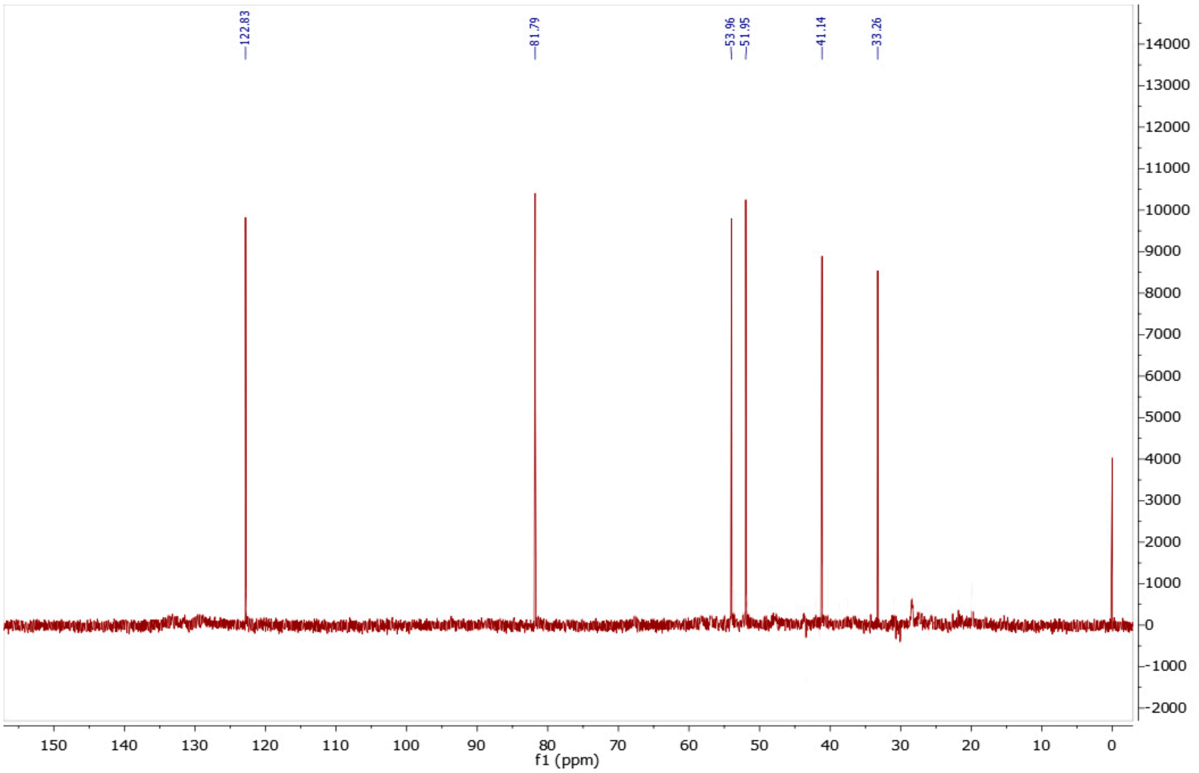

Figure S5: HSQC spectrum of **38** in $CDCl_3$.

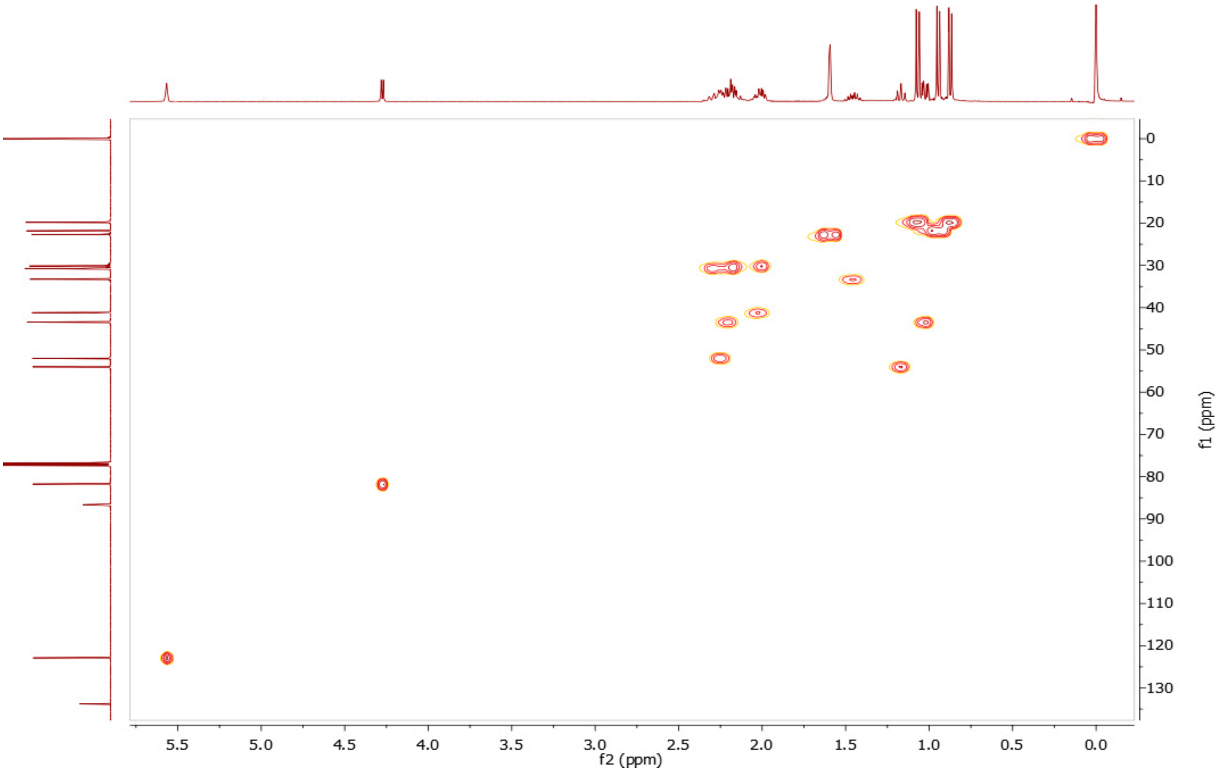

Figure S6: COSY spectrum of **38** in $CDCl_3$.

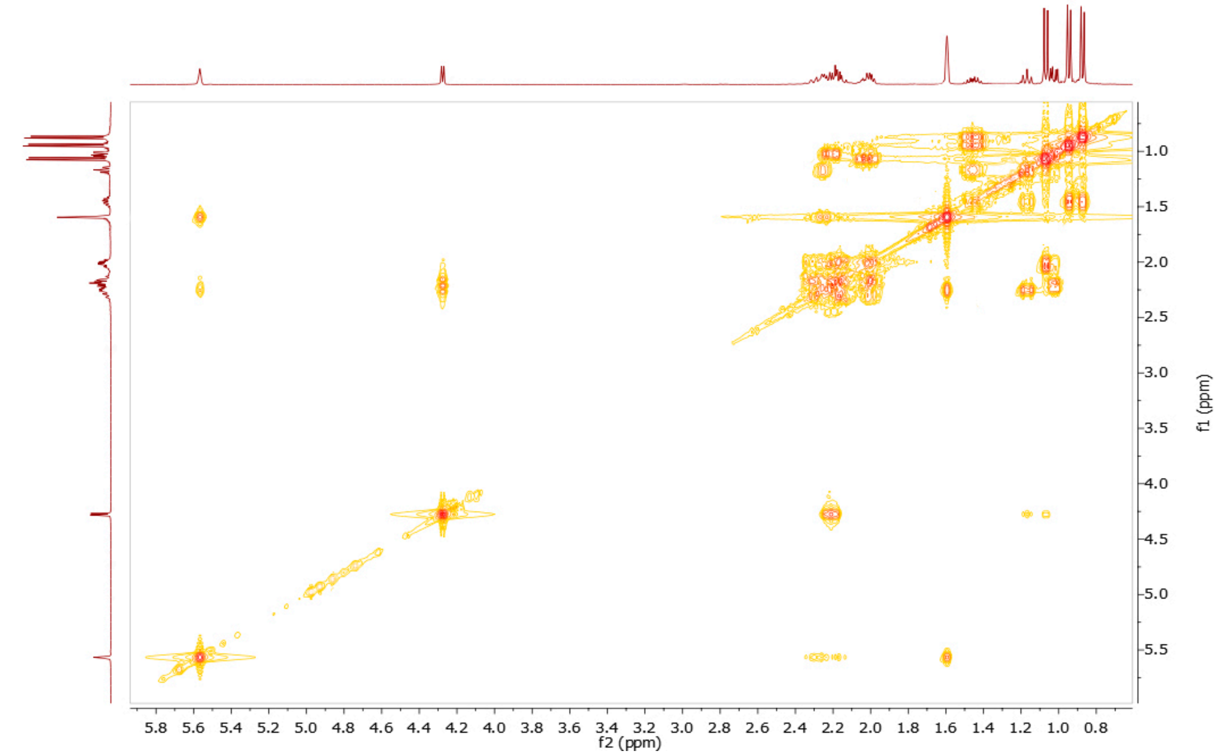

Figure S7: HMBC spectrum of **38** in $CDCl_3$.

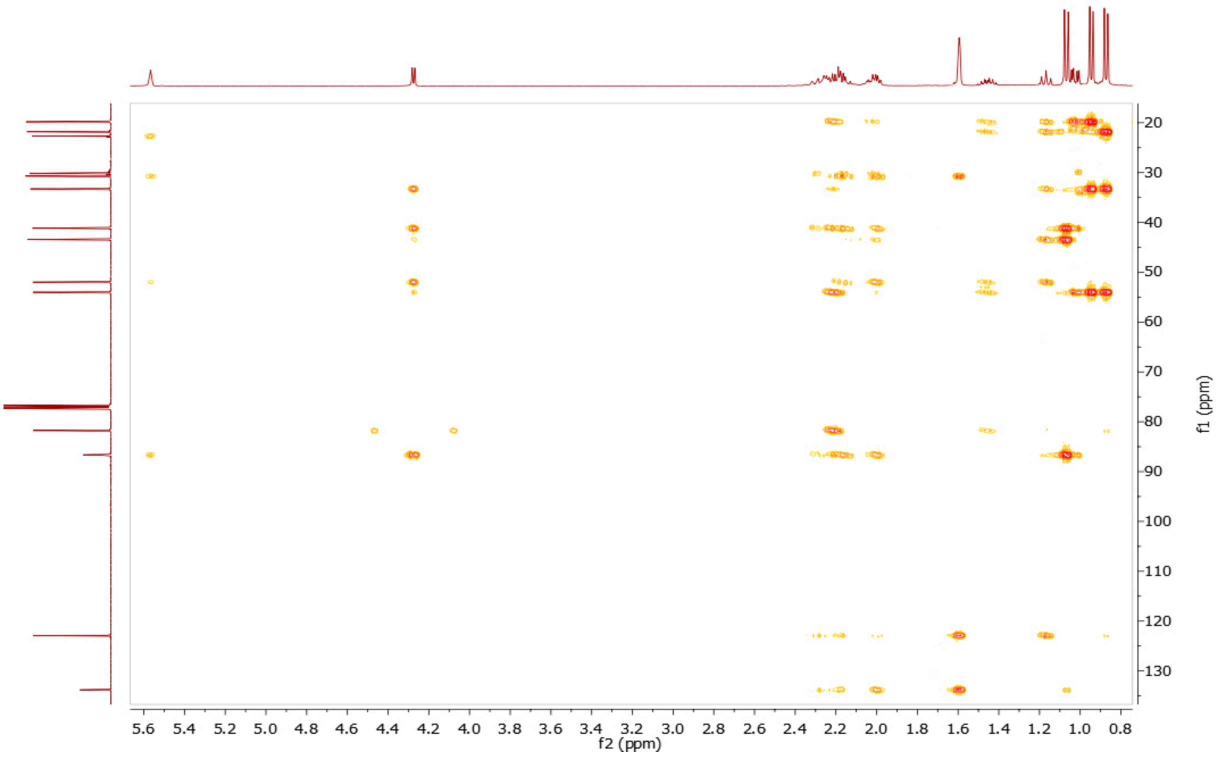

Figure S8: NOESY spectrum of **38** in $CDCl_3$.

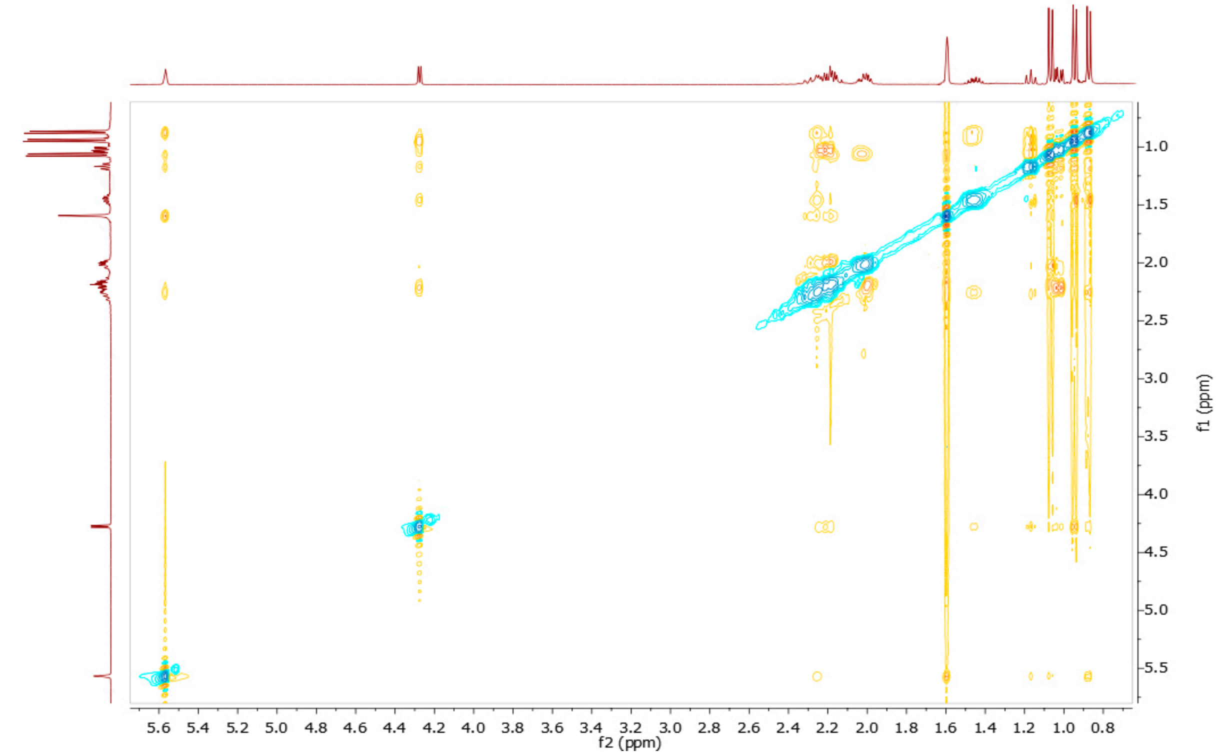

Figure S9: ^{1}H NMR spectrum of **52** and **53** in $CDCl_3$ (400 MHz).

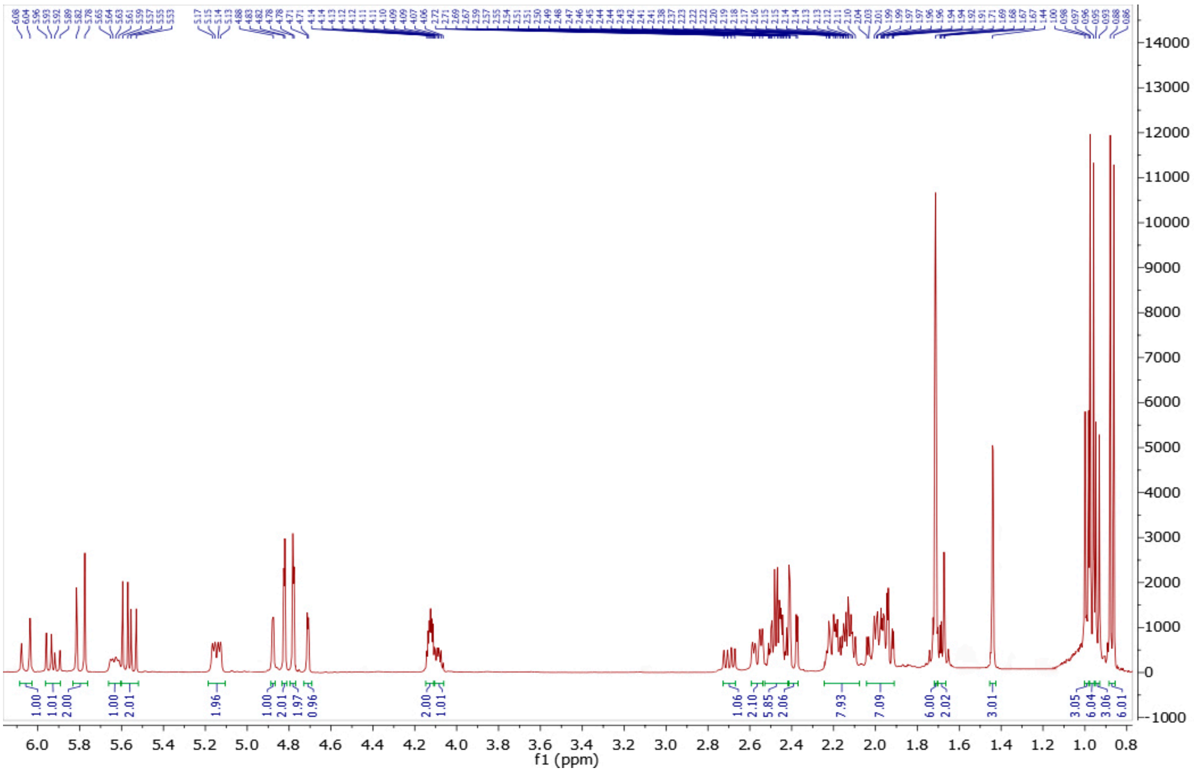

Figure S10: ^{13}C NMR spectrum of **52** and **53** in $CDCl_3$ (100 MHz).

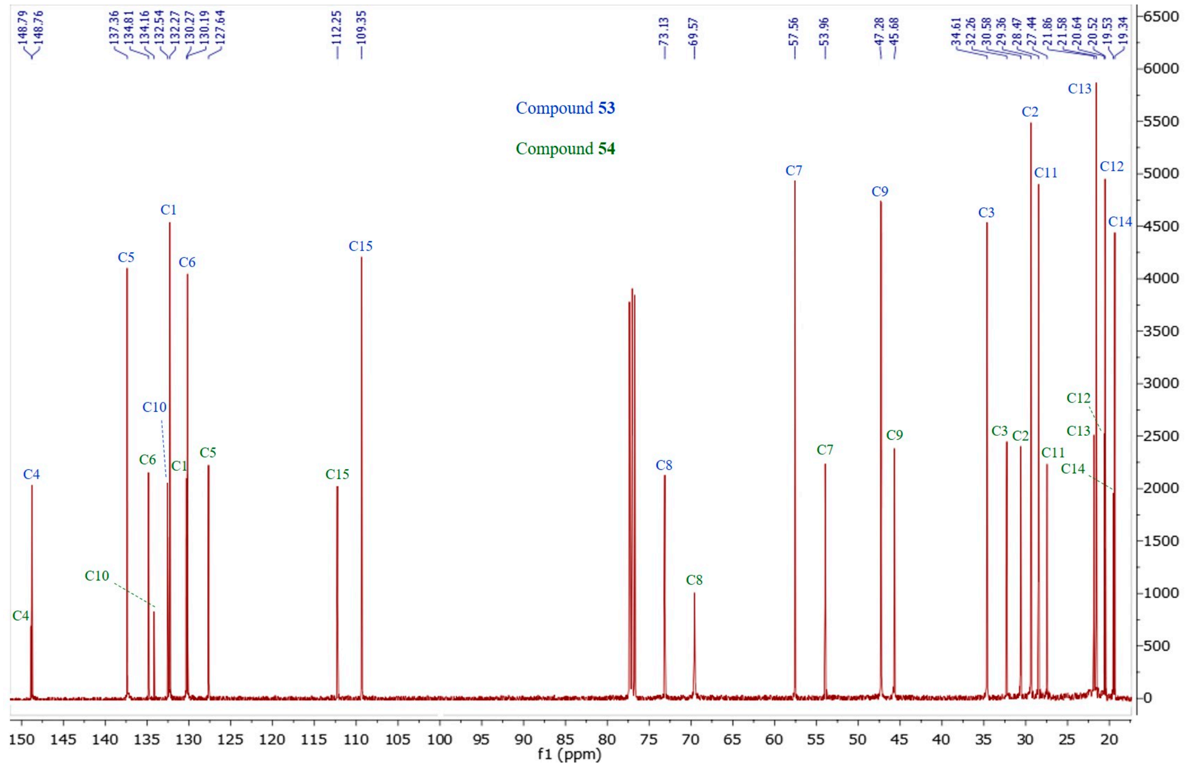

Figure S11: DEPT 135 NMR spectrum of **52** and **53** in $CDCl_3$ (100 MHz).

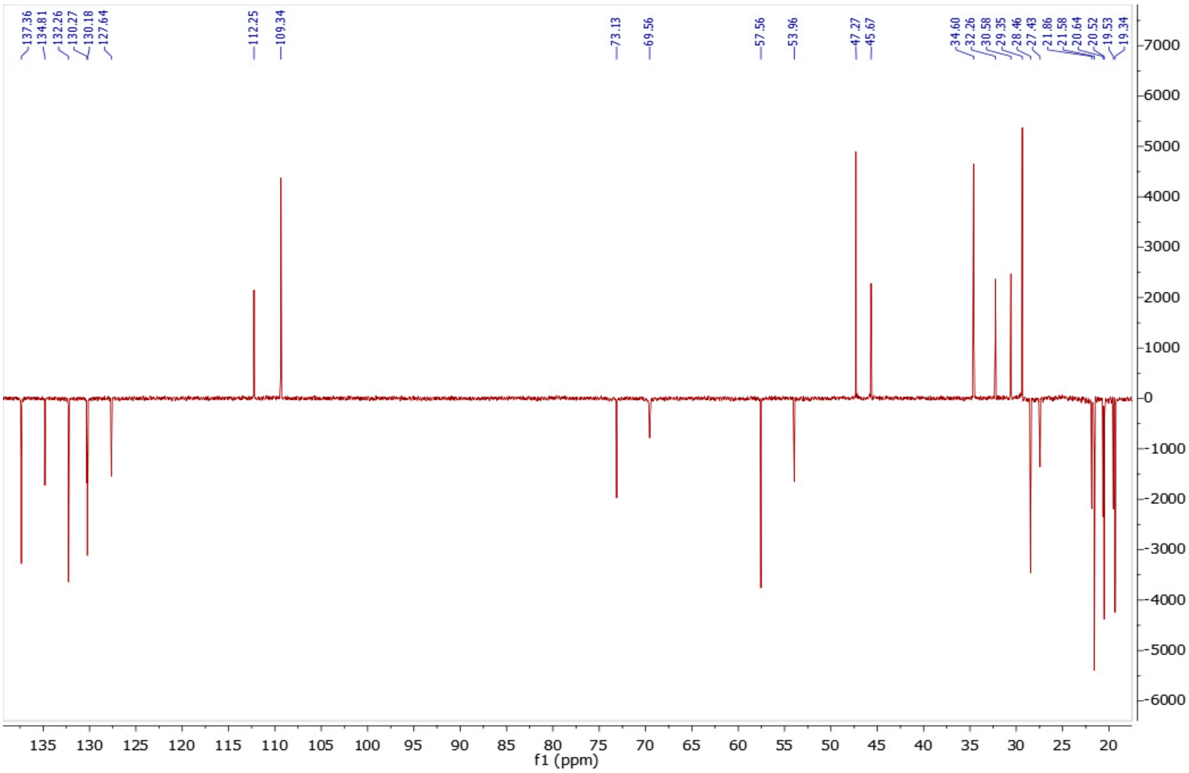

Figure S12: DEPT 90 NMR spectrum of **52** and **53** in $CDCl_3$ (100 MHz).

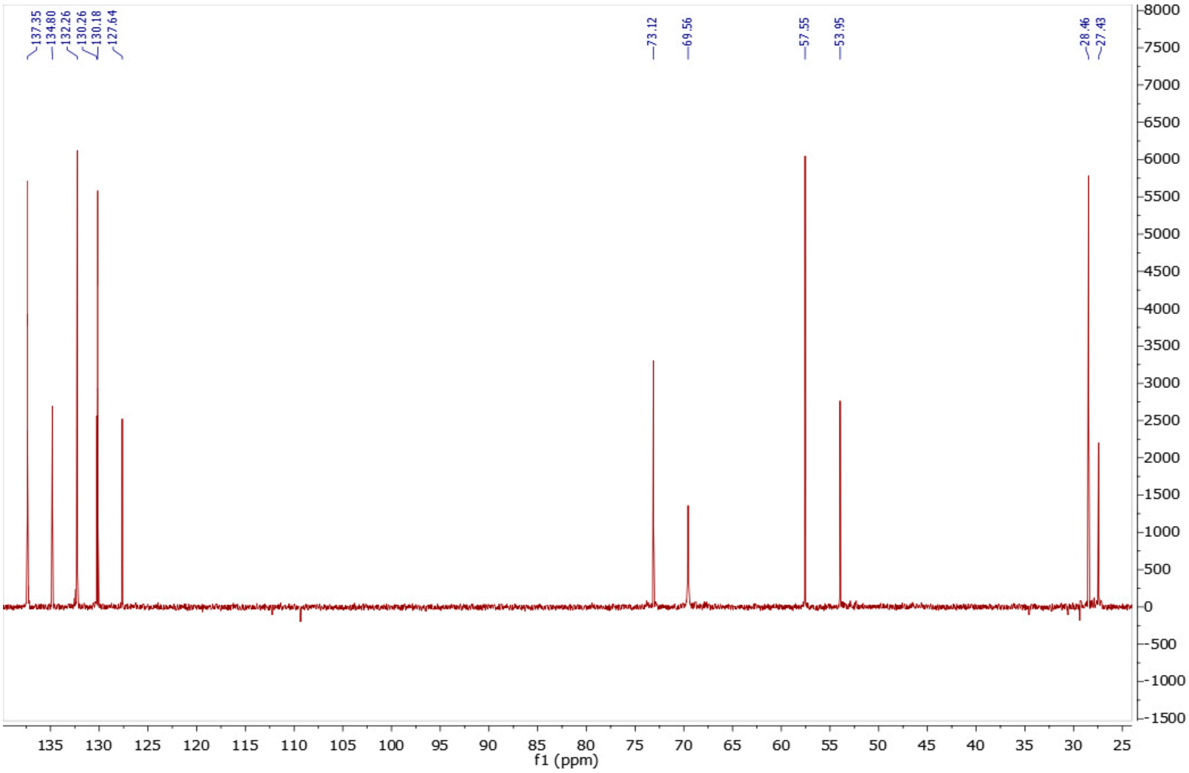

Figure S13: HSQC spectrum of **52** and **53** in $CDCl_3$.

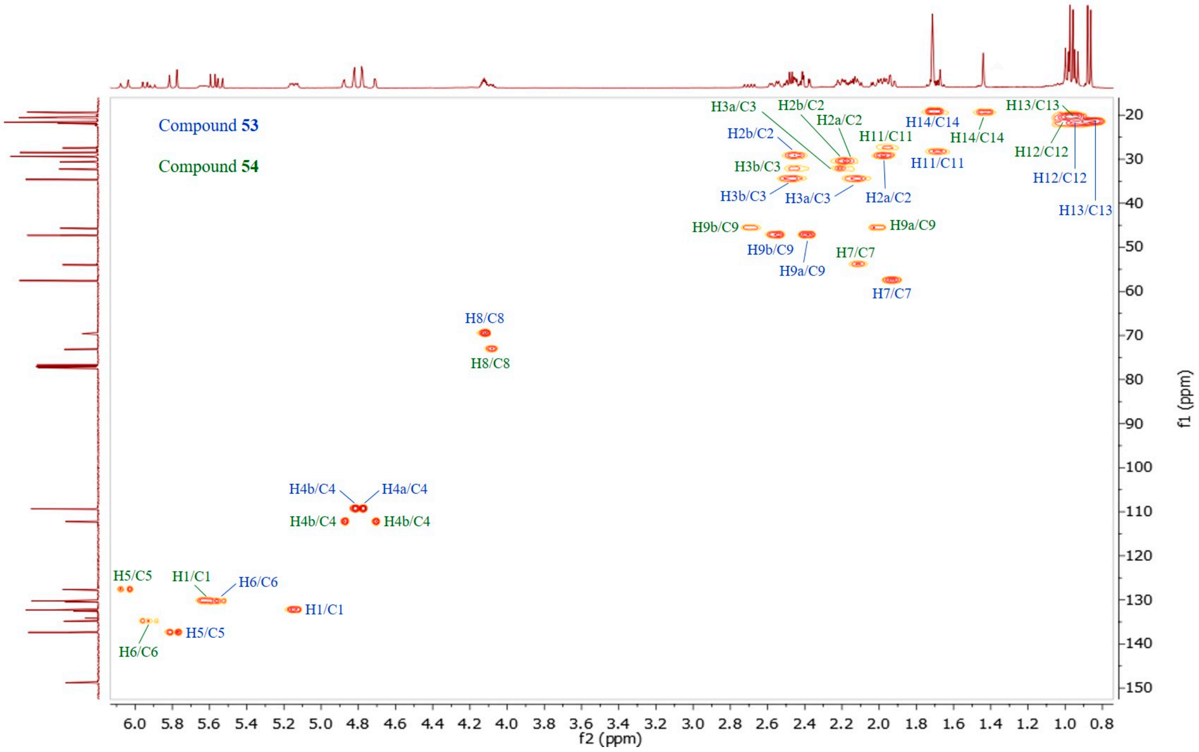

Figure S14: COSY spectrum of **52** and **53** in $CDCl_3$.

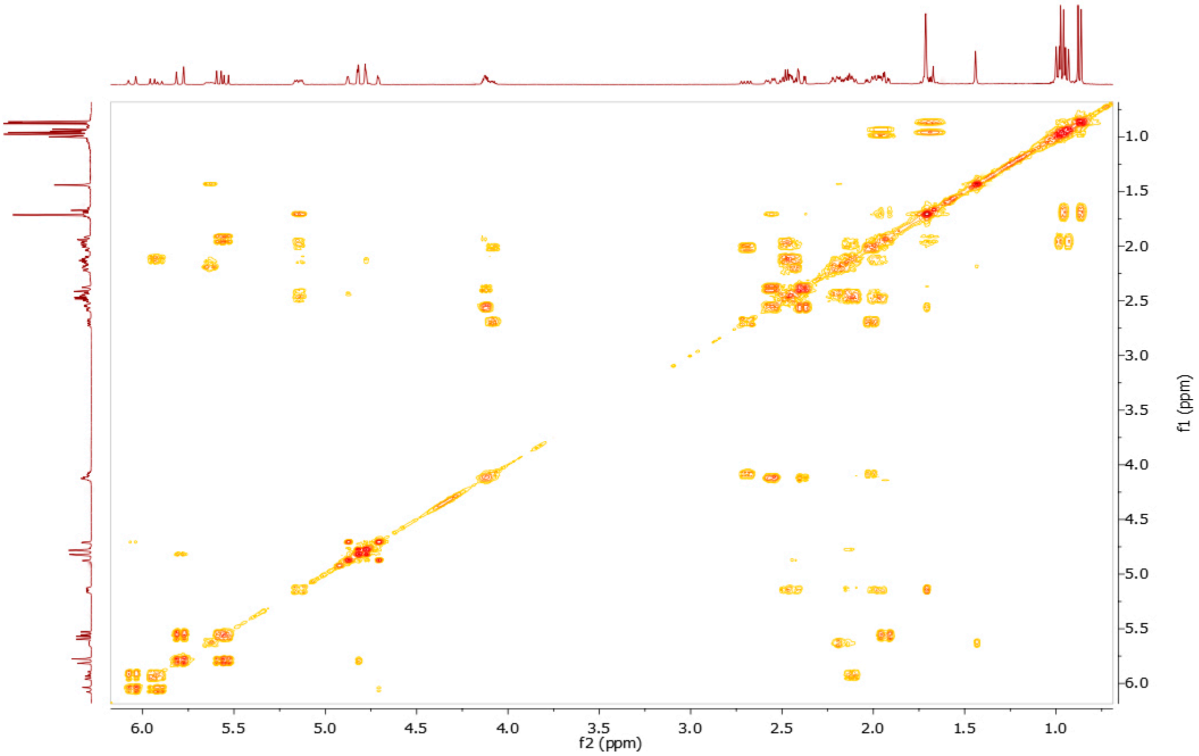

Figure S15: HMBC spectrum of **52** and **53** in $CDCl_3$.

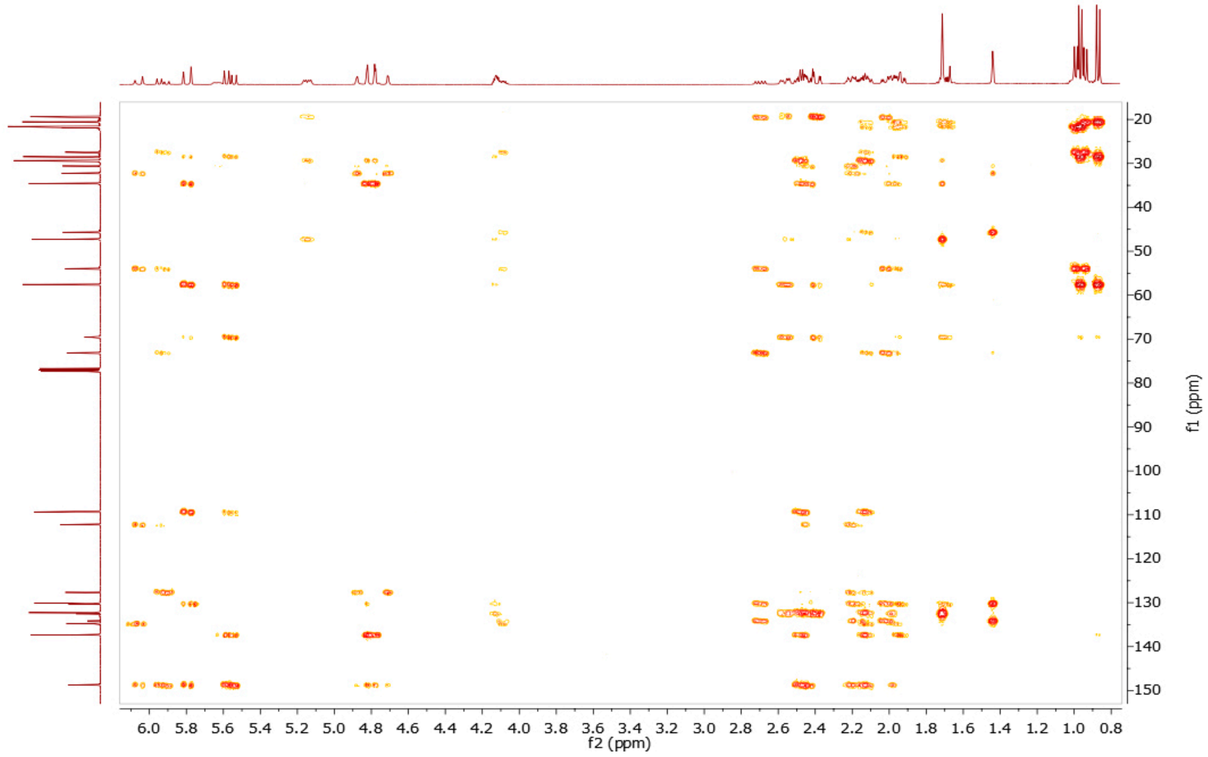

Figure S16: NOESY spectrum of **52** and **53** in $CDCl_3$.

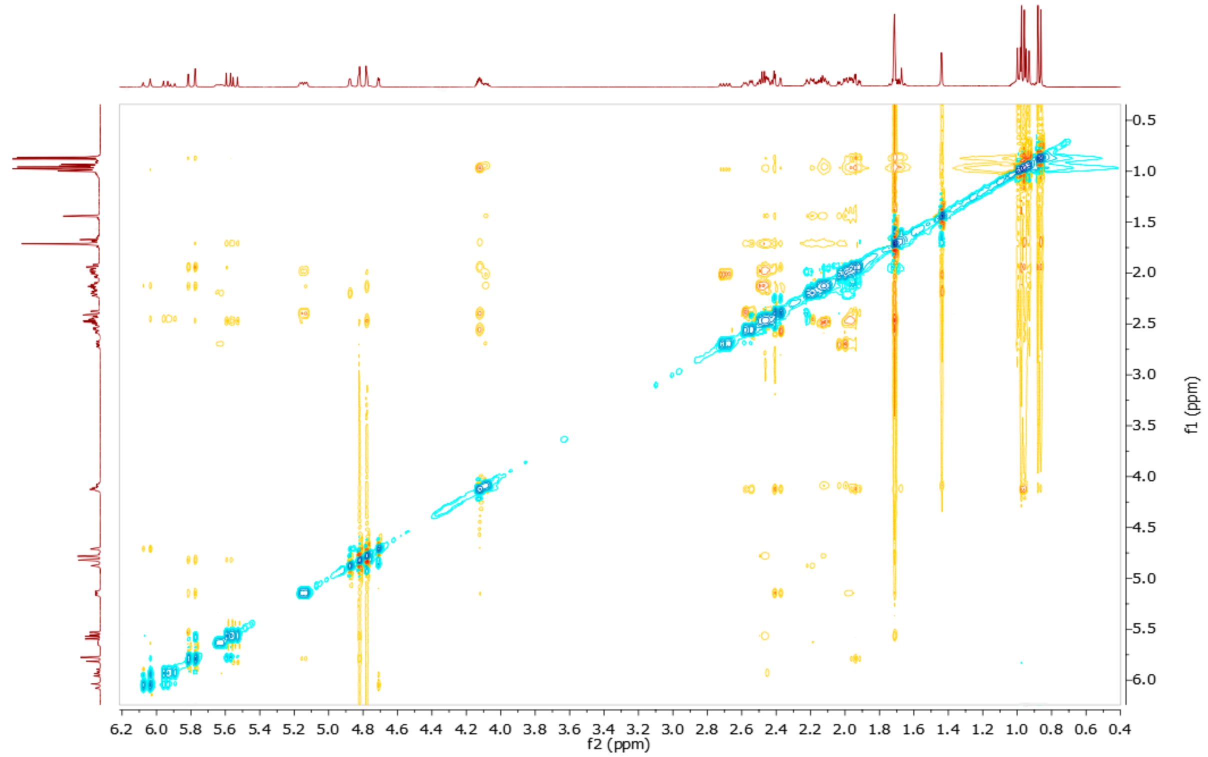

Figure S17: EI-MS spectra of **52** and **53**.

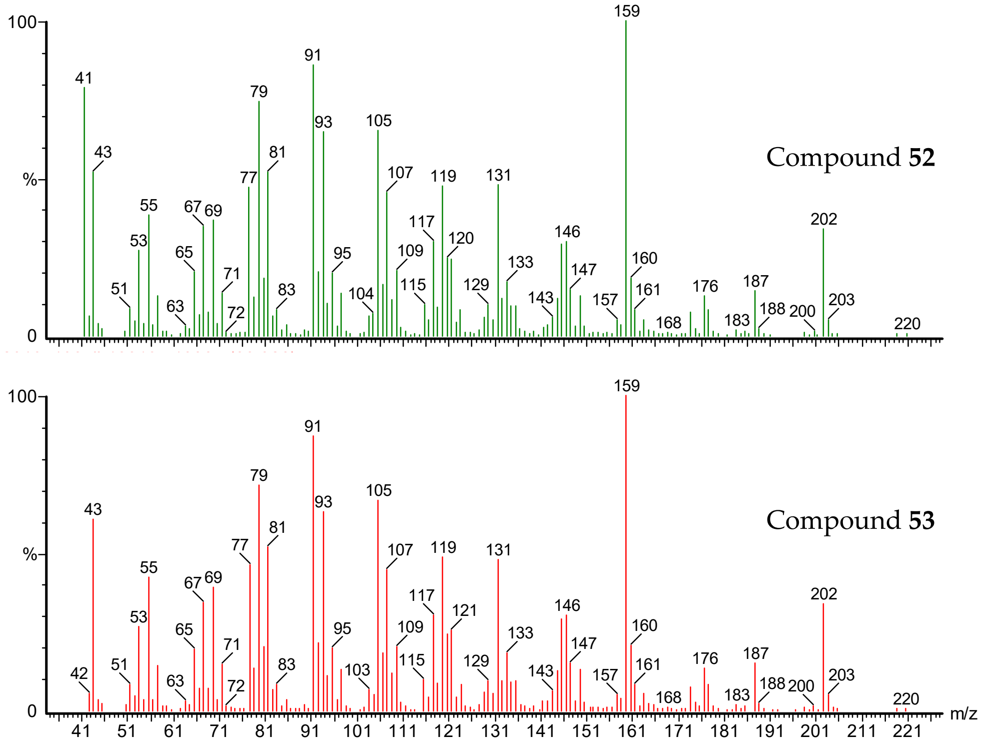

Figure S18: ^{1}H NMR spectrum of **56** in $CDCl_3$ (400 MHz).

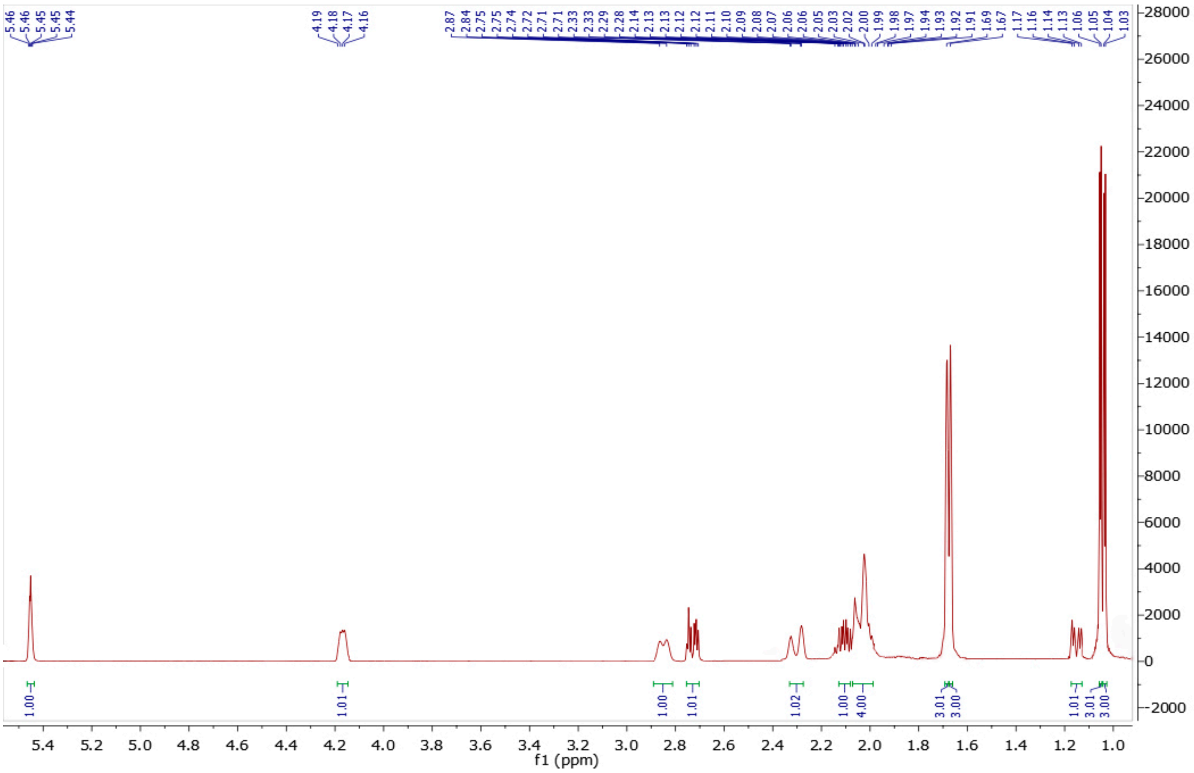

Figure S19: ^{13}C NMR spectrum of **56** in $CDCl_3$ (100 MHz).

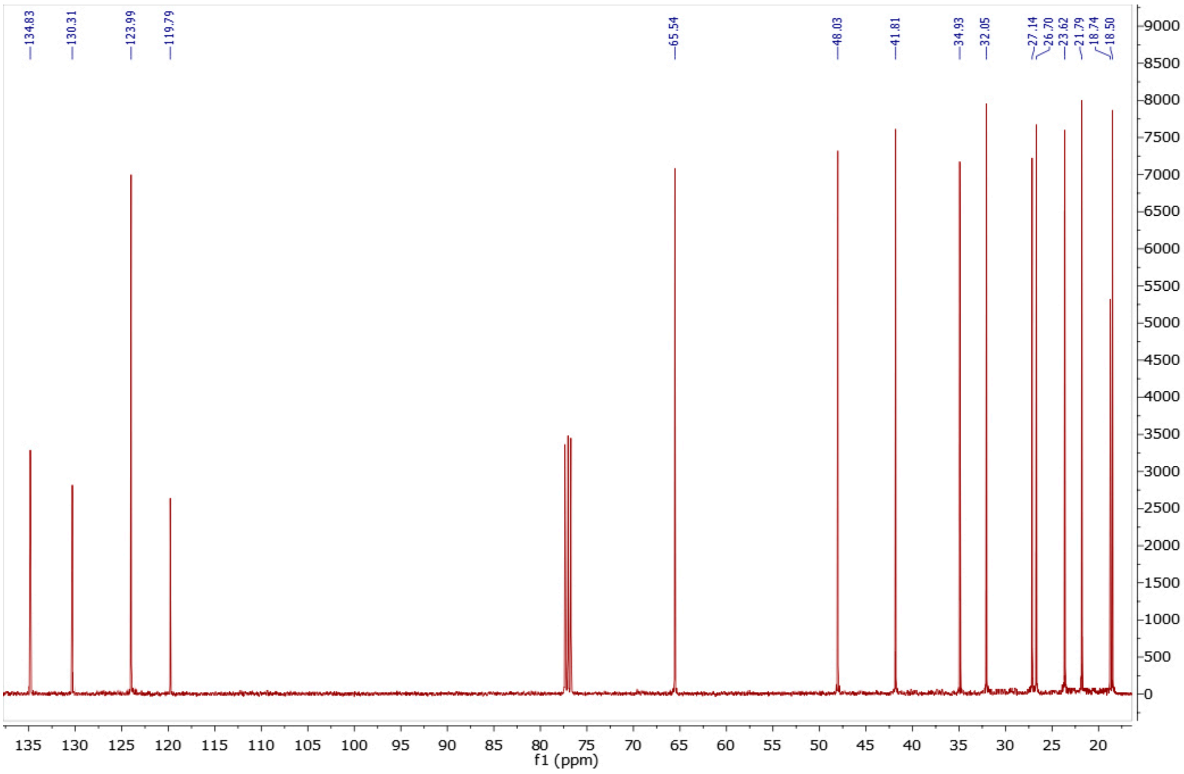

Figure S20: DEPT 135 NMR spectrum of **56** in $CDCl_3$ (100 MHz).

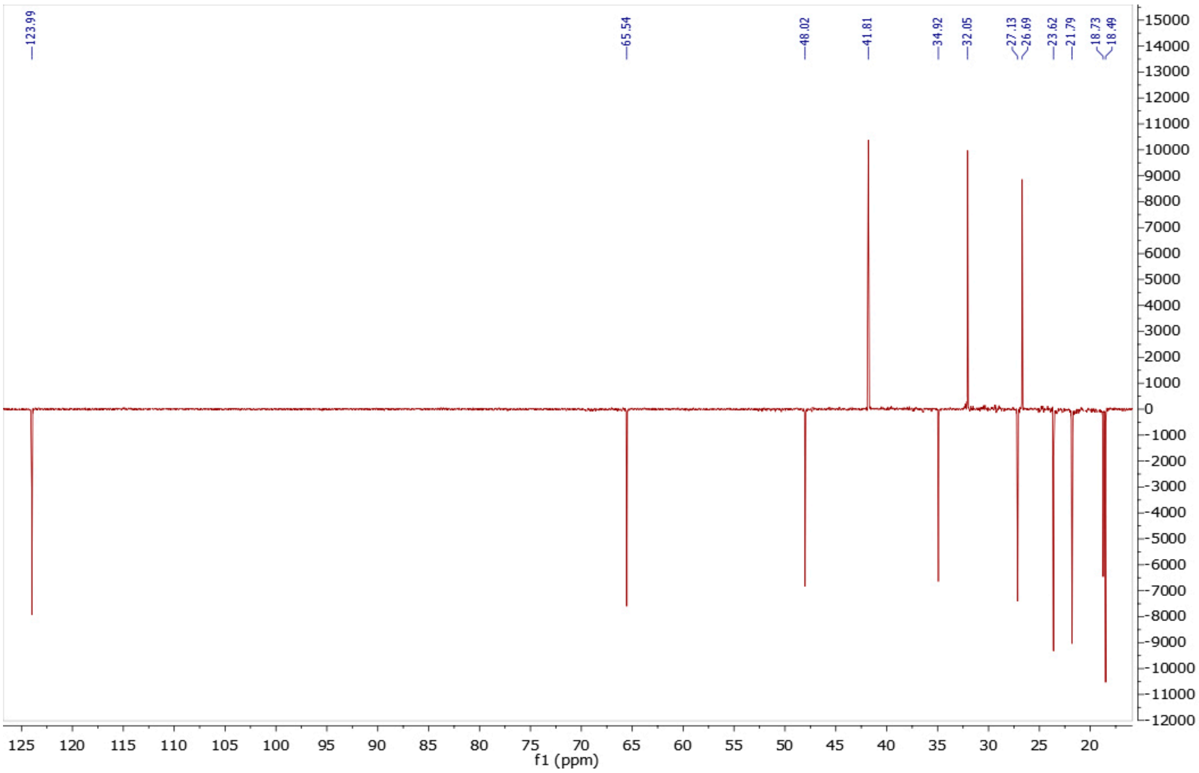

Figure S21: DEPT 90 NMR spectrum of **56** in $CDCl_3$ (100 MHz).

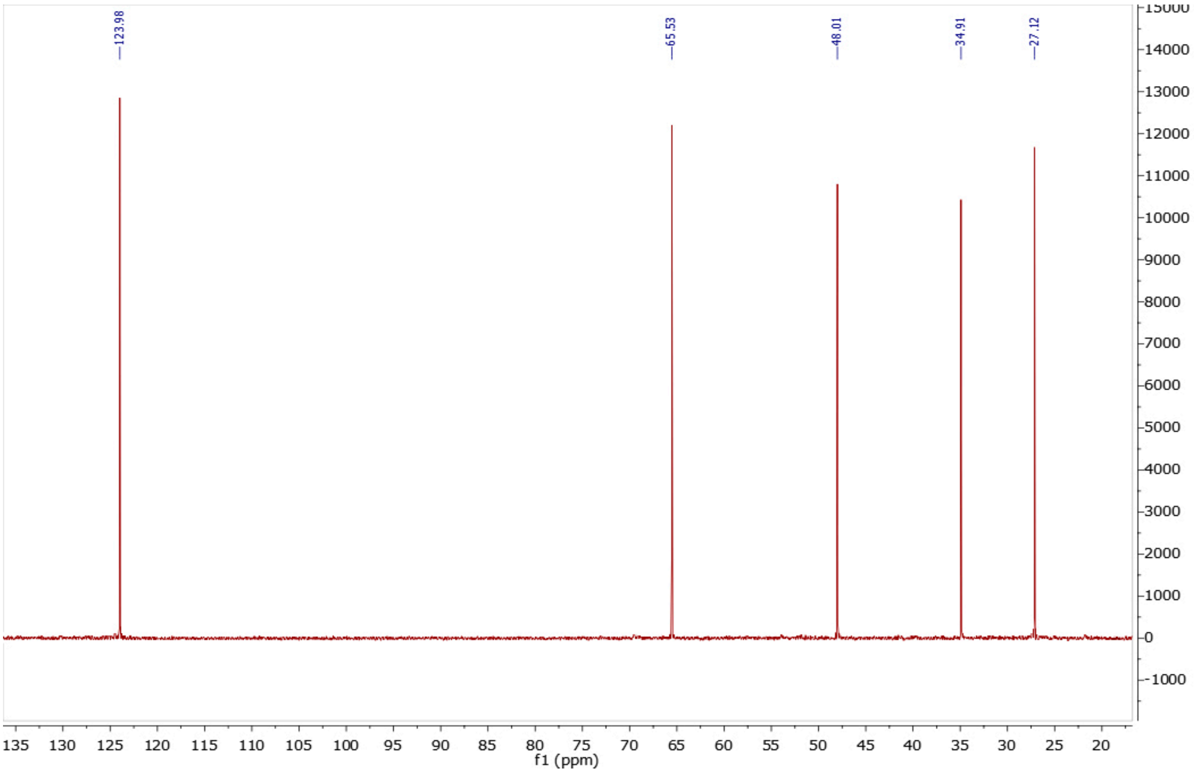

Figure S22: HSQC spectrum of **56** in $CDCl_3$.

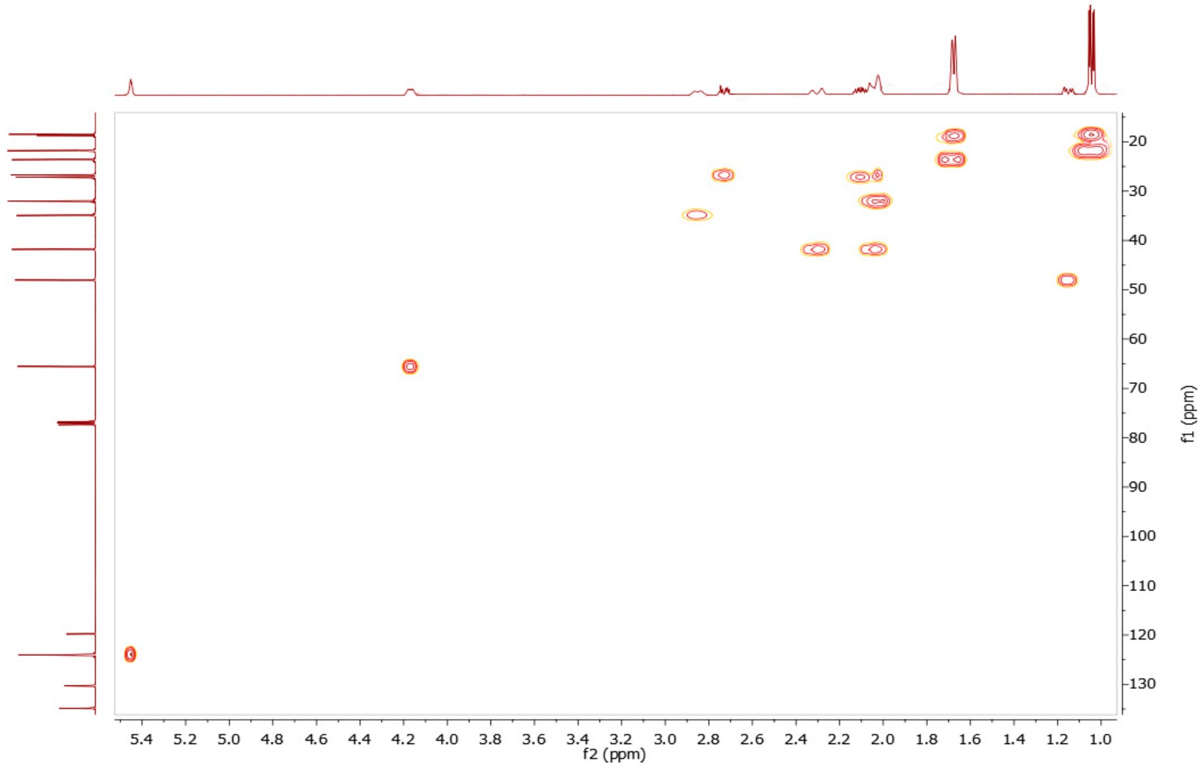

Figure S23: COSY spectrum of **56** in $CDCl_3$.

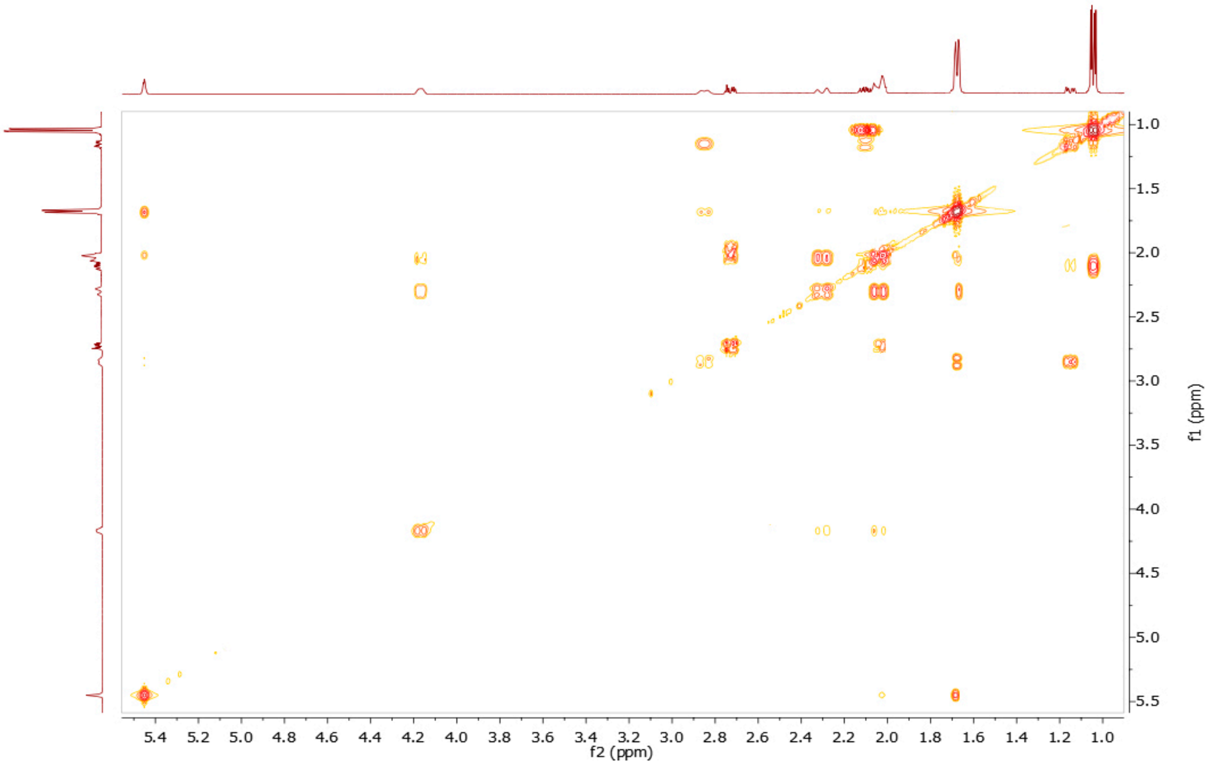

Figure S24: HMBC spectrum of **56** in $CDCl_3$.

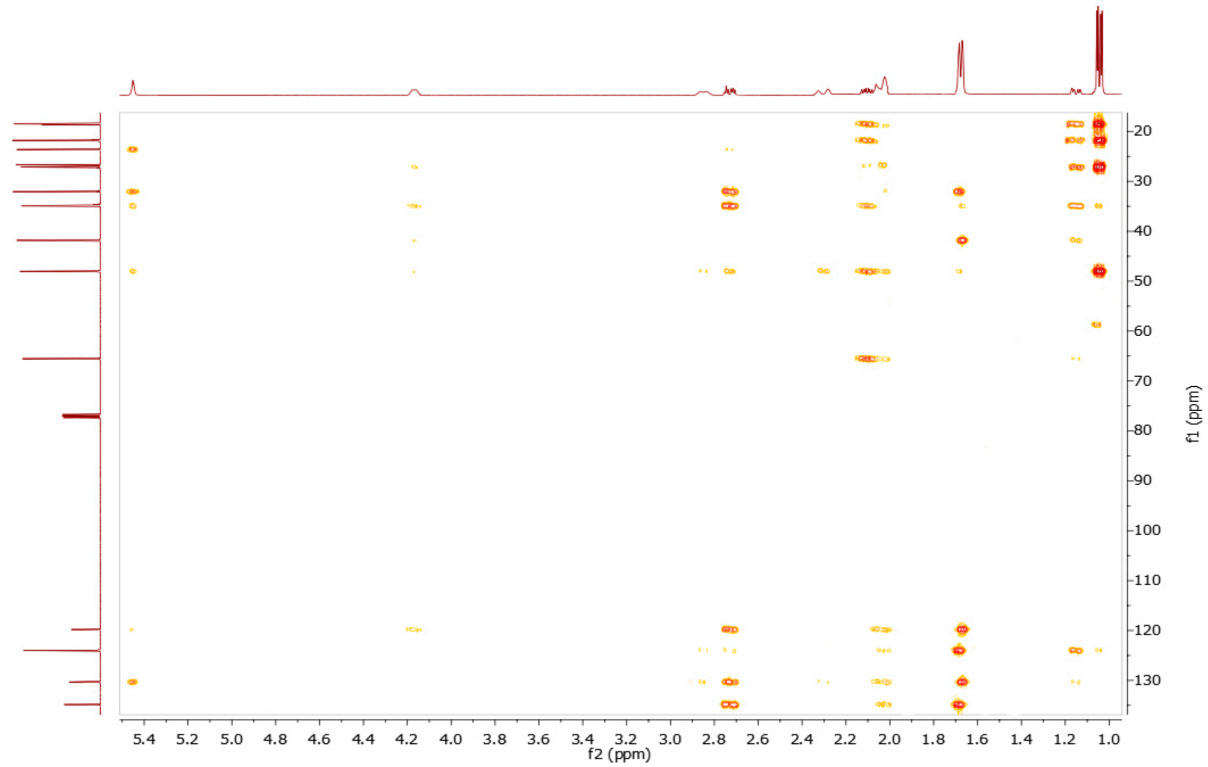

Figure S25: NOESY spectrum of **56** in $CDCl_3$.

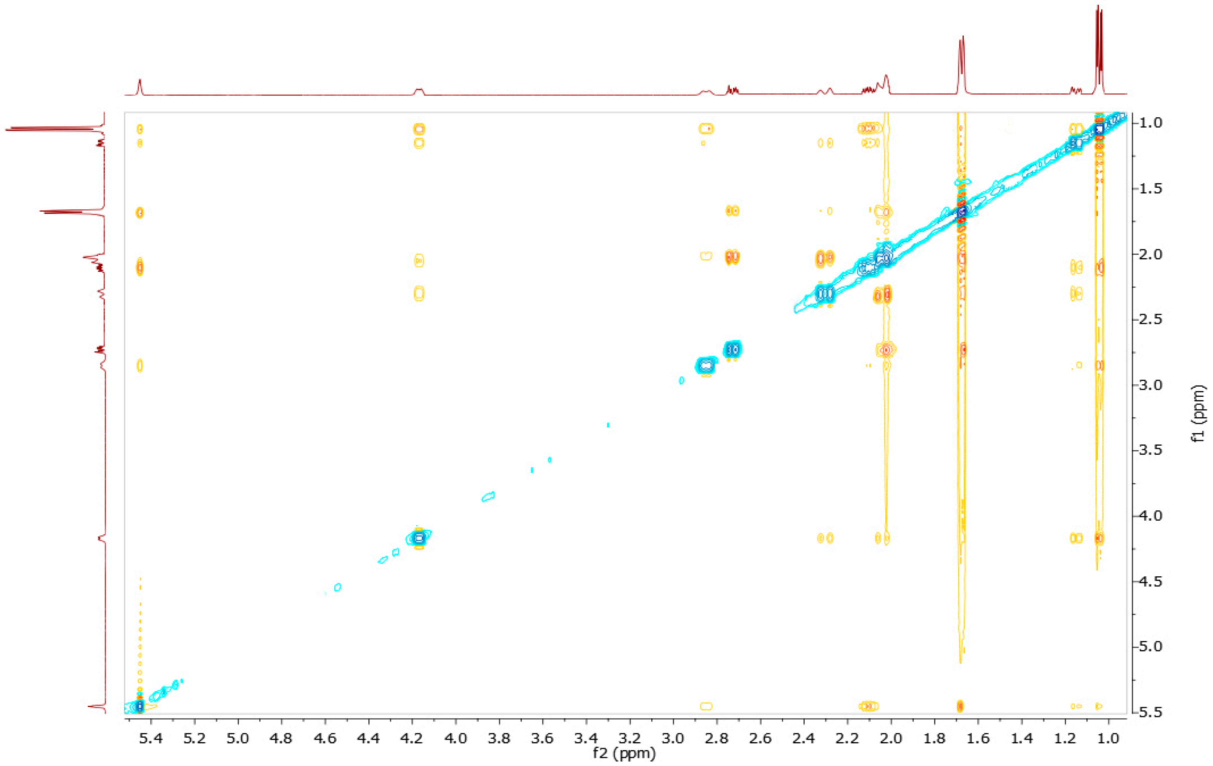

Figure S26: EI-MS spectrum of **56**.

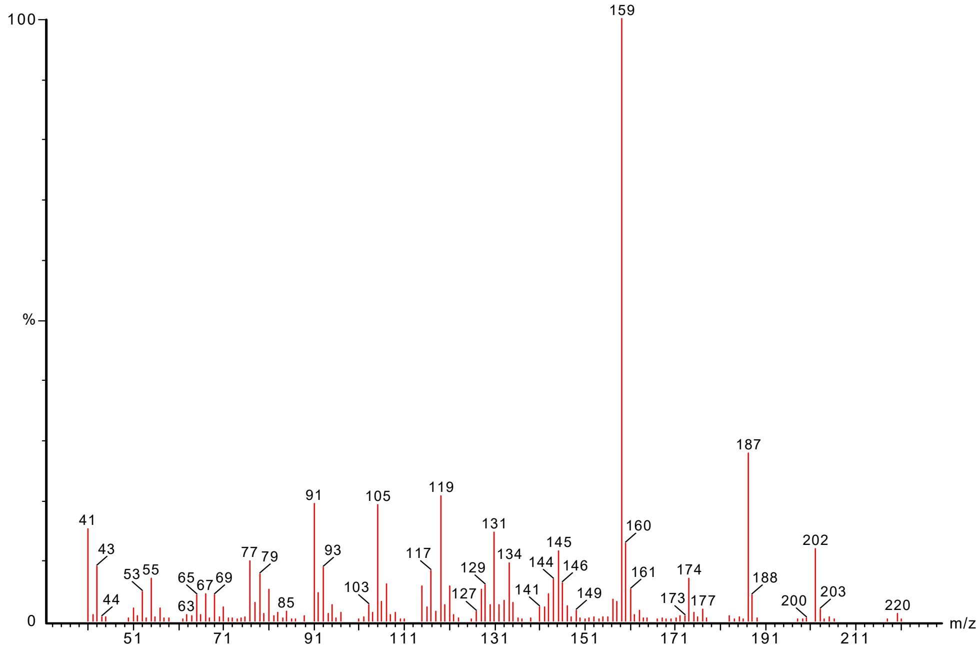

Supplement: Supplementary file 1 [file molecules-25-05613-s001.pdf]
